# Nursing staff competence assessment instruments: a scoping review with implications for long-term care

**DOI:** 10.1186/s12912-026-04755-0

**Published:** 2026-05-16

**Authors:** Juliane Mosenhauer, Sebastian Partsch, Ingrid Darmann-Finck

**Affiliations:** 1 Department of Health, Long-Term Care and Pensions, SOCIUM Research Center on Inequality and Social Policy, University of Bremen, Bremen, Germany; 2 High-Profile Area of Health Sciences, University of Bremen, Bremen, Germany; 3 Institute of Public Health and Nursing Research (IPP), University of Bremen, Bremen, Germany

**Keywords:** Nursing competence, Competence assessment instruments, Long-term care, Nursing homes, Healthcare assistants, Care aides, Dementia care, Palliative care, Scoping review, Competency frameworks, Workforce development

## Abstract

**Aim:**

Competence assessment is essential for professional growth, workforce planning, and quality improvement in long-term care (LTC). However, the wide range of instruments, their limited differentiation based on various qualification levels, and the lack of standardized frameworks hinder effective use. This scoping review aimed to map international evidence on competence assessment instruments used in residential LTC nursing, broadening the scope to include acute care, community-based, and mixed-care settings to enhance the relevance and transferability of findings.

**Methods:**

A scoping review was conducted following the Joanna Briggs Institute guidelines. Peer-reviewed and grey literature in English and German were searched across two databases (PubMed and CINAHL) between 2014 and 2024. Inclusion criteria focused on instruments assessing nursing competence through self-assessment or external evaluation, targeting various qualification levels and care contexts.

**Results:**

Twenty instruments were identified. The review addressed two key research questions: (1) A wide range of self-assessment tools with generally sound psychometric properties were found, predominantly developed for registered nurses and validated in acute settings; however, few differentiated by qualification level, and no validated instruments were identified for healthcare assistants. (2) Validated peer or external evaluation instruments were scarce, highlighting a reliance on self-perception and a lack of objective assessment. Instruments for specialized domains (e.g., dementia, palliative care) demonstrated higher contextual relevance but lacked robust testing in LTC.

**Conclusion:**

This review highlights significant variability and identifies a crucial gap in tools available for healthcare assistants and multi-method assessments. Future efforts should focus on creating modular, theory-informed, and context-sensitive instruments that are validated across various qualification levels and care sectors. These tools are essential to support individualized learning, ensuring equitable staffing, and fostering sustainable quality improvements in long-term care.

**Supplementary Information:**

The online version contains supplementary material available at 10.1186/s12912-026-04755-0.

## Background

In view of the ongoing transformation of healthcare systems driven by demographic changes, scientific progress, and an increasing complexity of care, continuous professional development has become indispensable for nursing staff [1]. It is not only essential for maintaining competence and motivation but also for ensuring patient safety and overall quality of care [2]. As care demands intensify, it is crucial to support nursing staff in evaluating and developing their competencies through structured approaches that link lifelong learning with clinical excellence.

To translate these overarching goals into everyday practice, nurses require structured approaches that help them evaluate their current performance, identify their learning needs, and pursue professional growth. Self-assessment of competence plays a central role in this process. It enables nurses and other nursing staff at various qualification levels to recognize their strengths and developmental needs, set individualized learning goals, and engage in tailored continuous education activities [3, 4]. Through guided reflection on knowledge, skills, and attitudes, self-assessment can strengthen professional identity and support ongoing learning processes [5].

The importance of such reflective mechanisms becomes evident when considering their documented impact on patient outcomes and workforce sustainability. Consistent professional development has been associated with reduced mortality and medical errors [6, 7] as well as increased motivation, engagement, and job satisfaction [8]. These factors contribute to staff retention and overall organizational performance [9]. This is particularly relevant in long-term residential care environments, where care is predominantly rendered to elderly adults living in nursing homes who often have complex health needs, such as multimorbidity, dementia, and palliative needs. Residential long-term care is distinguished by its delivery of continuous, around-the-clock services and an increasing escalation in care complexity. The workforce tasked with providing this care comprises registered nurses, healthcare assistants, and support staff with varying qualification levels, all of whom play integral roles in service delivery. Effective managerial support, particularly through relational leadership approaches, can facilitate the transfer of knowledge into practice and enhance the attractiveness of employment in these settings [10, 11]. It is important to recognize that competence development and assessment are relevant across all qualification levels. They are essential for ensuring safe, person-centred care and for creating coherent, qualification-sensitive development pathways.

Within this context, the **KomPers project** (“Competence-oriented Personnel Deployment in Nursing”^1^) funded by the Federal Ministry of Research, Technology, and Space, was launched in Germany. The project focuses on residential long-term care and aims to link competence assessment with personnel deployment and organizational development. Implemented in three academic nursing facilities, it seeks to establish a competence-based staffing model that integrates systematic competence assessment across qualification levels, targeted professional development, and organizational learning. It identifies qualification and training needs, develops workplace-based learning opportunities, and evaluates their effects on care quality and staff development. By aligning staff competencies with residents’ care requirements, KomPers contributes to strengthening professionalization, quality of care, and workforce sustainability in long-term care (LTC).

Competence assessment is central to achieving these goals. It serves both individual and organizational purposes:

- At the individual level, it supports reflection, learning, and career planning.
- At the organizational level, it provides evidence for targeted deployment, training strategies, and leadership development [12].
- At the professional level, it enhances the visibility, comparability, and standardization of nursing competencies, thereby contributing to the ongoing professionalization of the field [13, 14].

Although self-assessment is a widely adopted and valuable strategy for fostering self-reflection [15–17], it also has notable limitations, primarily, its inherent subjectivity. Moreover, self-perceived competence does not necessarily reflect actual performance [18]. Studies have shown that self-assessment tends to measure confidence more than competence [19, 20] and that discrepancies may arise between nurses’ and managers’ evaluations [21, 22]. Additionally, biases such as “faking good” or the tendency of less competent respondents to overestimate their abilities complicate interpretation [23, 24].

Because of these challenges, it is essential to combine self-assessment with complementary methods—such as peer review, structured observation, or knowledge testing—to ensure objectivity and comprehensiveness [25]. In this review, competence is defined as individual dispositions—such as knowledge, skills, and attitudes—that enable individuals to handle complex professional demands [26, 27]. This definition aligns with a “holistic conception of competence,” as derived by Cowan et al. [28] from their review of the literature. Instruments must therefore be carefully designed to capture this complexity while remaining practical, acceptable, and relevant across various qualification levels and care settings. Although the focus of this review is on residential LTC, instruments developed in other care settings may also provide valuable insights for competence assessment in this context.

Developing a valid and reliable assessment instrument requires a theoretical framework that clearly defines the construct of competence, in addition to yielding robust psychometric evaluation. According to Fitzpatrick et al. [29], sound instruments must demonstrate validity, reliability, and responsiveness to change to allow justified inferences about performance levels. However, studies have shown that only a limited number of instruments in nursing meet these standards [30, 31]. Given ongoing challenges such as workforce shortages, increasing care complexity, and the need for competence-based staffing, there is a clear demand for evidence-based guidance to inform practice and policy in LTC [32].

Although individual studies have examined specific competency assessment tools for nursing staff in detail, there is a lack of systematic comparative analysis. Systematic or scoping reviews that provide a broad overview of the various tools and their application contexts are rare [31]. To address this gap, this review uses established methodological frameworks for scoping reviews to systematically map and summarize the available evidence. Against this background, the present scoping review aimed to systematically map international evidence on competence assessment instruments relevant to nursing staff in residential LTC and to identify implications for their application in this setting.

Against this background, the present scoping review aimed to systematically map international evidence on competence assessment instruments used in residential LTC nursing, with a focus on identifying tools that are relevant, transferable, and adaptable to this context.

Specifically, this review seeks to answer the following research questions:

1. What self-assessment instruments exist, and what are their psychometric properties (e.g., reliability, validity) for nursing staff at various qualification levels, considering implications for long-term care?
2. What validated instruments of peer or external evaluation are available, and how do they complement self-assessment in capturing competencies across professional domains (e.g., clinical, interpersonal, and leadership) in the context of long-term care?

Through this review, we aim to identify and evaluate available instruments and derive implications for practice and policy. The findings will help nursing home managers and educators determine which instruments are most suitable for different contexts, identify gaps requiring new tool development, and propose ways to embed competence assessment into personal development plans, education continuation, and career pathways in LTC.

## Method

A scoping review was chosen because it is well-suited to systematically map international evidence on competence assessment instruments for various qualification levels used in residential LTC nursing, with a focus on identifying tools that are relevant, transferable, and adaptable to this setting. This approach also enables a structured appraisal of a broad and potentially heterogeneous body of literature, making it particularly suitable for the present study [33–35].

The systematization of this review followed the Joanna Briggs Institute (JBI) methodology recommendations for scoping reviews, a well-established and internationally recognized standard. The JBI approach is especially suitable for this review because it provides a structured framework for identifying, evaluating, and synthesizing the literature, thereby aiding in mapping the current knowledge on competence assessment tools [34]. In addition, this scoping review was conducted and reported in accordance with the Preferred Reporting Items for Systematic Reviews and Meta-Analyses Extension for Scoping Reviews (PRISMA-ScR) checklist [36] (see Related File 1 for the PRISMA-ScR-checklist) as well as the recommendations of Hirt and Nordhausen for systematic and transparent development and documentation of a literature search [37]. The integration of JBI guidelines and the PRISMA-ScR framework ensures methodological rigor, transparency, and reproducibility throughout the review process. While PRISMA-ScR supports the structured presentation of methods, search strategies, and results, the JBI methodology provides specific tools for data extraction, synthesis, and quality assessment [30]. The search procedure was conducted iteratively.

Since protocol registration is not mandatory [36], this review was not pre-registered. However, all stages—research question development, search strategy, data extraction, synthesis, and quality assessment—were systematically documented to ensure transparency, methodological integrity, and reproducibility [34, 37].

This review does not include a critical appraisal of the methodological quality of the included studies. This is consistent with the purpose of scoping reviews, which focus on mapping the breadth and nature of existing evidence rather than evaluating the validity or risk of bias of individual studies [34, 38]. The focus is on identifying, categorizing, and describing the types of instruments, their psychometric properties, and their applicability across contexts.

### Inclusion and exclusion criteria

The population – concept – context (PCC) scheme was used to specify the various search dimensions [33, 34, 38]. For this study, we focused on formal caregivers. This specification was chosen to include all formally qualified nursing staff in LTC, including registered nurses, nursing assistants with and without formal training, and exclude informal caregivers (e.g., relatives and friends).

The concept examined relates to the instruments used for competence assessment. These include self-assessment instruments or a combination of self- and external assessment instruments. In addition to instruments measuring general competencies, those addressing specific aspects of nursing care relevant to long-term care, such as palliative care, dementia care, gerontology, and patient safety, were explicitly included.

To ensure a sensitive search and to capture instruments that may not explicitly target long-term care but contain transferable competencies, the context was expanded to include acute inpatient and outpatient care settings. This decision was driven by the observation that many competency assessment instruments are developed in acute care contexts but contain content highly relevant to long-term care (e.g., palliative care, patient safety, dementia care). By including these contexts, the review aims not only to map existing instruments but also to identify cross-contextual competencies and derive implications for long-term care practice.

However, upon closer reflection and during the data extraction process, it became clear that the instruments used in nursing education (Bachelor’s and Master’s programs) primarily assess competency acquisition during training, rather than professional competency in practice. Since this study focuses on assessing competencies of practicing nurses, not those developed in academic or training settings, nursing education contexts were excluded.

To ensure methodological rigor and transparency, the inclusion and exclusion criteria were iteratively refined through team consensus. After screening the first 100 articles, a team meeting was held to discuss discrepancies and to revise the criteria. The following adjustments were made [39]:

- **Population**: All formally qualified nursing staff in long-term care settings were included, regardless of their formal training status.
  Specialist-trained nurses (e.g., geriatric psychiatrists, clinical nurse specialists) were excluded to focus on general nursing competencies; informal caregivers remained excluded.
- **Concept**: Validation studies, translation studies, and cross-cultural adaptation studies of competency assessment instruments were specifically included because they offer essential evidence on instrument reliability, validity, and transferability across different contexts and cultures. Instruments that assess professional competence in specific domains—such as palliative care, dementia care, communication, gerontology, pain management, and patient safety—were explicitly included.
  Instruments that assess self-efficacy, integrative care, or mental health as standalone constructs were excluded because they measure psychological or emotional traits rather than professional nursing competencies. Additionally, evaluation, simulation, observational, and interview-based studies of training programs or individual courses were excluded because they focus on training processes or program impact rather than on validated competency assessment instruments. Competency assessments related to care or therapeutic concepts (e.g., “therapeutic relationship” as a standalone construct) were excluded to ensure conceptual clarity.
- **Context**: The scope was expanded to include acute inpatient and outpatient care settings. Nursing education (Bachelor’s/Master’s programs), training programs, simulation studies, and evaluation studies of individual courses were excluded because they evaluate competency development during training rather than professional competence in practice. Specialized settings—such as psychiatric nursing, emergency care, intensive care, surgical nursing, and pediatric nursing—were excluded because instruments used in these contexts typically assess highly specialized, setting-specific competencies rather than generalizable nursing competencies relevant to long-term care. Additionally, pure knowledge tests (like multiple-choice quizzes) were excluded because they do not assess professional competence. Exceptions were made for tools that integrate knowledge within broader competency frameworks or focus on LTC-relevant areas such as dementia, palliative care, or geriatrics. This approach ensures that the review includes practice-oriented, context-specific competency assessment tools.

These refinements were documented in a decision log and applied consistently in the remaining screening and extraction process [37].

The final inclusion and exclusion criteria are summarized in Table 1, which details the initial and updated criteria across the PCC dimensions, along with the rationale for changes.

**Table 1:** Final inclusion and exclusion criteria of the scoping review: iterative refinement based on the PCC framework and team consensus

PCC-Dimension	Initial Criterion (A priori)	Inclusion Criteria (Post-Pilot)	Exclusion Criteria (Post-Pilot)	Rationale for Change
**Population**	Nursing staff at various qualification levels; **excluded informal caregivers**	Nursing staff at various qualification levels (e.g., registered nurses, nursing assistants with or without formal training)	Informal caregivers, Specialist-trained nurses (e.g., clinical nurse specialists)	To focus on general nursing competencies in practice, not specialized medical roles
**Concept**	Instruments assessing nursing competencies via self- or peer assessment, supervisor assessment, surveys, qualitative or quantitative methods; **excluded psychological assessment tools and simulation-based evaluations**	Competence assessment, measurement, self-assessment and peer assessment, questionnaires, surveys, Qualitative and quantitative methods assessing professional competence, validation studies, translation studies, cross-cultural adaptation studies of competency assessment instruments, Competence assessments on topics such as palliative care, dementia care, communication, gerontology, pain, patient safety	Psychological assessment tools, generic knowledge tests, Simulation-based evaluations, Competency assessments related to care or therapeutic concepts, Instruments assessing self-efficacy, integrative care, or mental health as standalone constructs, Observational, or interview-based studies of training programs or individual courses	To ensure focus on professional nursing competencies and validated instruments; exclude studies assessing training processes or psychological traits; include studies providing evidence on instrument reliability, validity, and transferability
**Context**	Long-term care settings; nursing education, Nursing studies (Bachelor’s and Master’s programs); **no exclusions**	Long-term care, Acute inpatient care, Outpatient care	Psychiatric nursing, Nursing education (Bachelor’s and Master’s programs), Emergency care, Intensive care, Surgical nursing, Pediatrics	To focus on generalizable, transferable competencies; exclude instruments assessing setting-specific competencies or training/evaluation processes

### Data sources and searches

Two electronic databases were independently searched by two reviewers: PubMed/MEDLINE (JM) and CINAHL (SP) for publication records. These two databases are considered the most relevant databases for biomedical, nursing, and allied health research. In accordance with the methodological guidance for scoping reviews [33], which recommends conducting an initial search in at least two relevant databases to identify key concepts and refine the search strategy. These databases were chosen for their complementary strengths. PubMed offers extensive coverage of biomedical and clinical literature, while CINAHL provides specialized indexing for nursing and allied health disciplines, including qualitative and practice-based research, which is often not included in other databases. Since this review focuses on competence assessment instruments in nursing and residential LTC, these two databases were considered suitable for capturing the relevant literature.

Only studies published in English or German were included in this review. This language restriction was mainly due to the research team’s language expertise, which ensured reliable interpretation, accurate data extraction, and consistent evaluation of the psychometric properties and cultural adaptation of competency assessment tools. Because these instruments are sensitive to linguistic and cultural differences, maintaining methodological rigor required that all included studies be accessible in languages that the authors were fully proficient in.

The online research covered the period from January 2014 to November 2024. To identify instruments that are used in current nursing practice or research, and thus have a high degree of applicability to LTC, the search period was limited to the last ten years. An example of a full search string for PubMed/MEDLINE and CINHAL is presented in Table 2.

**Table 2:** Complete search string of the database search in PubMed/MEDLINE and CINHAL

database	Search string	Results
PubMed / MEDLINE	„((((NURSES[Mesh] OR “NURSING STAFF”[Mesh] OR “NURSE PRACTITIONER*“[Title/Abstract] OR „LICENSED PRACTICAL NURSE*“ OR “registered nurse*“[Title/Abstract] OR “nurse assistan*“[Title/Abstract] OR “PRACTICAL NURSE“[Title/Abstract])) AND ((“SELF-ASSESSMENT”[Mesh] OR “SURVEYS AND QUESTIONNAIRES”[Mesh] OR „Evaluation Studies as Topic“ [Mesh] OR “competence SCALE*“[Title/Abstract] OR “skill scale*” OR “insight scale” OR “self-reflection“[Title/Abstract] OR “NURSING COMPETENCE SCALE"OR “COMPETENCY ASSESSMENT“[Title/Abstract]))) AND ((“PROFESSIONAL COMPETENCE“[Mesh] OR “CLINICAL COMPETENCE“[Mesh] OR “CORE COMPETENCIES” OR “NURSE COMPETENCE” OR “nursing abili*”))) AND ((“LONG-TERM CARE“[Mesh] OR “NURSING CARE“ [Mesh] OR “NURSING HOME“[Title/Abstract] OR “RETIREMENT HOME“[Title/Abstract])) AND ((humans[Filter]) AND (english[Filter] OR german[Filter]) AND (y_10[Filter]))“	273
CINHAL	(TI nurs* OR AB nurs* OR MH Nurses + OR TI nurse assistant# OR AB nurse assistant#) AND (TI competenc* OR AB competenc* OR MH competence OR MH clinical competence OR TI professional competence# OR AB professional competence# OR TI core competence# OR AB core competence# OR TI ablilit* OR AB abilit* OR TI skill# OR AB skill# OR MH nursing skills) AND (TI assess* OR AB assess* OR TI competenc* N3 assessment OR AB competenc* N3 assessment OR MM Competency Assessment OR TI measur* OR AB measur* OR TI self-assessment OR AB self-assessment OR TI self-evaluation OR AB self-evaluation OR TI self-reported OR AB self-reported OR TI self-reflection OR AB self-reflection OR TI questionnaire OR AB questionnaire OR TI scale OR AB scale OR MH research instruments) AND (AB “long term care” OR TI “long term care” OR MH nursing homes + OR MH long term care nursing OR MH residential care)	555

### Selection process

**Fig. 1:**
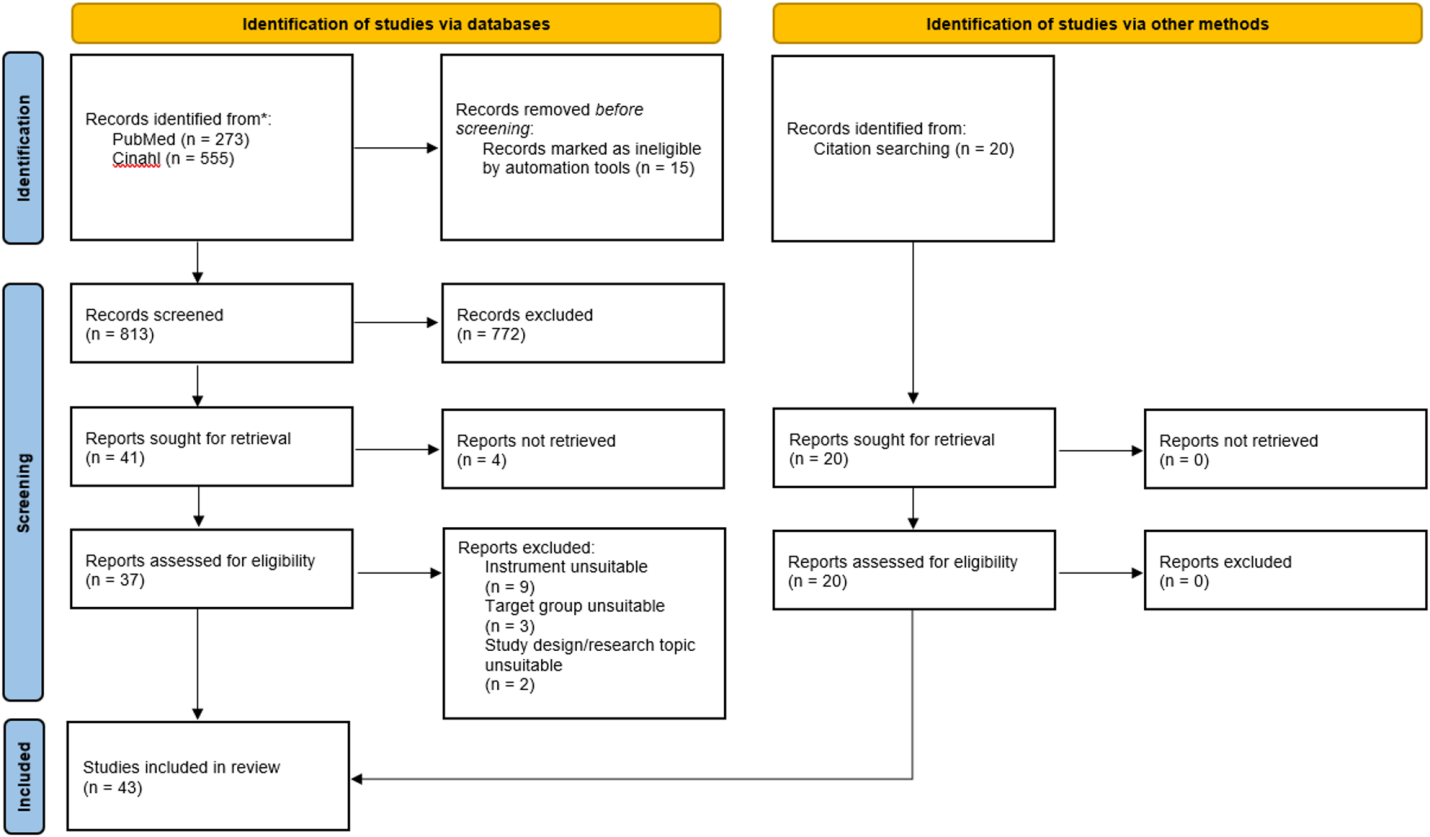
PRISMA flow diagram of the selected articles for review [44]

The free version of the AI-powered platform Rayyan was used to compile the relevant literature, identify duplicates, and screen titles and abstracts [40]. Title/abstract screening was initially conducted blind by both authors (JM and SP). After screening the first 100 articles, all authors met to discuss discrepancies and to reach consensus on the inclusion and exclusion criteria and the data extraction process, guided by questions regarding missing criteria, redundant criteria, and unclear extraction items. After screening the first 100 articles, all authors convened to discuss discrepancies and reach a consensus on the inclusion and exclusion criteria and the data extraction process. They focused on questions about missing criteria, redundant criteria, and unclear extraction items. Following this team meeting, JM and SP independently re-evaluated all 100 articles from the initial phase using the updated criteria. This re-evaluation helped finalize the inclusion and exclusion decisions based on the refined criteria. Subsequently, the remaining articles were screened using the final version of the criteria. The entire selection process was documented in a PRISMA-ScR flow diagram (see Fig. 1 in the Results section), which details the number of records identified, screened, assessed for eligibility, and included in the final dataset. Title and abstract screening continued with the final criteria (Table 1), and the results were reviewed collaboratively. To assess inter-rater reliability, Fleiss’ kappa (κ) was calculated to measure the agreement between multiple raters in categorical assessments, considering random agreement [41]. Inter-rater reliability was calculated using Fleiss’ kappa, showing a relatively high level of substantial agreement among the raters (κ = 0.63) [42]. Articles selected by both reviewers were automatically included. Articles selected by one reviewer were discussed based on the inclusion and exclusion criteria, and inclusion or exclusion was determined. In cases of uncertainty or disagreement, a third reviewer (IDF) was consulted to reach a consensus. This process ensured that the refined criteria were applied consistently and transparently across the screening phase. Articles were excluded if the target group of the study did not correspond to the question posed in the scoping review, the research topic was deemed unsuitable, or the target group or environment was inappropriate for answering the research question. All exclusions were documented to ensure transparency and traceability of the selection process.

### Data extraction

After reviewing and analyzing the titles and abstracts, the authors formed initial inductive categories based on the identified literature. These categories were derived from the main topics of the included studies and served as a preliminary structure for the instruments [33]. The two researchers independently extracted the data from the included articles.

Subsequently, the categories were supplemented and condensed using content analysis based on Mayring’s approach [43]. The initially formed categories were reviewed and expanded through systematic comparison, summarization, and revision using MAXQDA (Version 2020, JM / Version 2022, and SP) software. This approach enabled new content aspects of the instruments from the articles to be integrated and existing categories to be refined and anchored theoretically.

During the extraction process, the two authors held regular discussions to reflect on the progress and ensure that the category system captured all information relevant to answering the scoping review questions [33]. We also checked the bibliographies of each study and used existing networks and organizations to identify additional relevant studies [33, 36]. These networks consisted of experts in the fields of nursing education and nursing science. Five experts were contacted through personal conversations based on their institutional affiliations and recognized contributions to the field of LTC. All had substantial expertise in the clinical aspects of nursing care. Their methodological expertise varied: one expert had experience in scoping reviews, one in competency assessment instruments and nursing education, and three had a background in nursing science.

The resulting category system for the instruments was thus developed through an iterative process of inductive category formation and content-analytical structuring to ensure the transparent and comprehensible consolidation of content-related results.

The final categories used for the comparative analysis of the instruments covered the following topics:

- **Diversity of target groups and professional roles**: This category examined the target groups for which the instruments were used. The focus was on differentiation within professional nursing; for example, between nursing staff with different levels of qualifications.
- **Settings and fields of application**: This category covers the context in which the data were collected. An analysis was conducted to determine whether the instruments were used or tested in residential LTC, in various nursing settings, or exclusively in specific areas of practice.
- **Competence foci**: This category describes the competence aspects covered by the instruments. While some instruments aim to measure general nursing skills, others focus on specific competencies in care or activities within nursing practice.
- **Instrument structure**,** categories**,** and response formats**: This category examined the structure of the instruments—whether they contained subcategories and the number of items provided for each dimension, and the response formats used, such as Likert scales, dichotomous items, or open-ended questions.
- **Mode of data collection: self- versus external assessment**: This category describes the type of data collection. A distinction was made between instruments based on self-assessment by participants, peer assessment by third persons, supervisor assessment, or a combination of both procedures.
- **Validation and psychometric properties**: This category includes only those psychometric quality criteria explicitly reported in the studies and directly relevant to the research questions.

These categories formed the fundamental basis for a comparative analysis of the identified instruments, enabling their content and methodological characteristics to be presented in a structured manner.

## Results

A total of 828 publications were identified through database searches, with an additional 20 supplementary records found via citation searches (backward citation tracking of included articles). After removing duplicates using Rayyan software, 837 articles proceeded to title and abstract screening. Of these, 61 articles met the criteria for full-text review. Ultimately, 43 articles were included in the analysis (Fig. 1) [44].

The selected studies involved 20 competence assessments conducted across 23 countries. Most were cross-sectional (*n* = 19). Instruments were sourced from development and psychometric evaluation studies (*n* = 14) and from secondary research involving the adaptation, application, or psychometric evaluation of existing tools (*n* = 7) (see Supplementary Table S1). Ten of the included studies were published outside the search period, but were retained as they developed or validated the instruments. They were identified via citation searching to capture the original conceptualization and psychometric basis, even if published earlier. This diverse evidence base was intentionally included to systematically map international competence assessment instruments currently in development, validated, or used in nursing research and practice.

The instruments were categorized by setting and field of application into four groups: LTC (*n* = 9), acute inpatient care (*n* = 6), and mixed settings (*n* = 5). The majority of the 20 instruments were self-assessment tools (*n* = 18) with reported psychometric properties (*n* = 20). The instruments used a variety of response formats. Most instruments used 4- to 5-point Likert scales (*n* = 12). The Professional Nurse Self- Assessment Scale of Clinical Core Competencies (ProffNurse SAS) used a 10-point Likert scale [3], and the Holistic Nursing Competence Scale (HNCS) used a 7-point Likert scale [45]. Five instruments combined Likert scales with dichotomous items (Nursing Older People Competence Evaluation Tool - NOP-CET [25], Palliative Care Survey - PCS [46], Dementia Care Competence Scale - DCCS [47]), open-ended questions (Educational Needs Assessment - ENA [48]), or a visual analogy scale format (Nurse Competence Scale - NCS [16]). Only the Knowledge-about-Older-Patients-Quiz (KOP-Q) use only dichotomy “true or false” [49]. The number of domains in instruments varies significantly, ranging from 2 to 11 domains, and the number of items ranged from 13 to 346, indicating a broad spectrum of length and complexity. The instruments targeted diverse groups (see Supplementary Table S2), and some studies combined self-assessment with external evaluations (*n* = 2); however, how these methods complement each other in assessing competencies across clinical, interpersonal, and leadership domains remains unclear.

The presentation of the research findings is structured according to the main analytical categories used in this review: target groups and professional roles; competence foci and Instrument structure, categories; mode of data collection; and psychometric properties (see Supplementary Table 2). This categorization enables a structured comparison of instrument applicability and supports the identification of transferable competencies across care contexts.

### Target groups and professional roles

The instruments included in this review were primarily designed for registered nurses and other nursing staff with formal qualifications, such as nursing-home nurses (*n* = 10). A subset of instruments addressed mixed nursing staff with varying qualifications, including nurses and care assistants working together in the same setting (*n* = 4) and nurses and nurse students (*n* = 2). Additionally, four instruments targeted multiple professional groups, such as interdisciplinary teams comprising nurses, doctors, physiotherapists, and social workers. Notably, no instruments were identified that exclusively focus on healthcare assistants or care aides, whether with or without formal training. This gap highlights a significant limitation in the current evidence base, particularly given the central role of these staff in LTC settings.

### Competence foci and instrument structure, categories

The reviewed instruments differ considerably in their conceptual focus. Some measure general professional competence, whereas others capture specific palliative care, dementia care, or patient-centered skills. The instruments were structured using various frameworks, ranging from multidimensional models to domain-specific constructs, and vary significantly in their structure.

#### General professional competence foci

Eight instruments assessing broad, overarching competencies applicable across nursing roles and settings using multidimensional frameworks.

- **Commonalities Across Instruments**
  Despite differences in structure and context, the instruments assessing general professional competence share common thematic domains such as clinical practice, ethical behavior, communication, and professional development.
  These domains are consistent with established frameworks such as Benner’s “From Novice to Expert” [16, 28], which emphasize holistic, multidimensional approaches to professional competence.
  However, the specific content and operationalization of these domains vary across instruments. Similar to the NCS and Nurse Professional Competence (NPC) Scale, the European Healthcare Training and Accreditation Network (ETHAN) Questionnaire Tool is based on a holistic competence framework that integrates knowledge, skills, values, and professional attitudes. The HNCS integrates both general and professional domains, including ethical practices, teamwork, and professional development [45]. Rather than emphasizing a specific clinical domain, it captures broad professional competence dimensions relevant to all nursing contexts, thereby contributing to the conceptual understanding of competence as a multidimensional construct.
- **Variation by setting**
  The structure and focus of these instruments differ significantly across care settings. Instruments for LTC settings emphasize holistic, person-centered care and long-term relationship management. Domains include ethical practice, communication, cooperation, clinical care, safety, and professional development (ENA [48], Nurse Competence in Care Home Scale - NCCHS [50], Long-Term Care Nursing Competence Scale - LTCNC Scale [51]). Instruments for Acute inpatient care settings focus on rapid clinical decision-making, technical skills, and team-based care. Domains include clinical practice, ethical decision-making, leadership, and professional development (HNCS [45], ProffNurse SAS [3], ETHAN Questionnaire Tool [52]). Instruments used in mixed settings are the NCS and the NPC Scale. The NCS is mostly used in hospitals, but is also applied in all healthcare settings [53]. The NPC Scale has been developed and tested in general hospitals, community settings, and specialized units [54]. These differences in variation by setting on structure and focus suggest that the conceptualization of general professional competence depends on the context, even though the main domains are similar across settings.
- **Target groups**
  Licensed nurses are the primary target group in six of the eight instruments (ENA [48], NCS [16], NCCHS [50], HNCS [45], LTCNC Scale [51], ProffNurse SAS [3]). Two instruments (ETHAN Questionary Tool [52] and NPC Scale [54]) also include nursing students.

#### Specialized competence foci

The 20 included instruments were categorized by competency focus, with 12 instruments measuring specialized competencies in domains such as gerontology, palliative care, dementia care, and patient-centered care. These instruments are designed for specific clinical contexts and professional roles, reflecting the complexity of nursing practice in various settings.

- **Commonalities Across Instruments**
  Despite differences in focus and context, all specialized instruments share a domain-specific structure, with domains typically organized around knowledge, skills, attitudes, and behaviors.
  In palliative care, the Palliative Care Competence Framework Questionnaire (PCCF) and Palliative Care Nursing Self-Competence Scale (PCNSC) evaluate competencies in specific skills, such as physical, psychological, and spiritual needs [55, 56]. The Bonner Palliative Knowledge Test (BPW) combines cognitive knowledge testing with self-efficacy assessment in palliative practice [57], and the PCS combines palliative care practice with knowledge and skills [46]. The gerontological instrument focuses on clinical, ethical, and leadership skills in caring for older adults (Gerontological Nursing Competencies Scale - GNC Scale) [58]. Dementia care instruments address knowledge, skills, and attitudes in managing cognitive and behavioral challenges (DCCS, Sense of Competence in Dementia Care Staff - SCIDS) [47, 59]. Competence in person-centerd care is captured by the Patient-centred Care Competency (PCC) Scale [60], Individualized Care Scale-Nurse (ICS-Nurse) [61], and the Person- Centered Care Assessment Tool (P-CAT) [62]. They measure person-centeredness and organizational support for individualized care.
- **Target Groups**
  Instruments assessing specialized competencies (e.g., palliative care, dementia care, gerontology) are more likely to target multiple professional groups or nursing staff with varying qualification levels, reflecting the interdisciplinary and team-based nature of specialized care.
  Of the 12 instruments with specialized competence foci, eight addressed multiple professional groups (PCCF [55], BPW [57], SCIDS [59], P-CAT [62]) or mixed nursing staff with different qualification levels (KOP-Q [49], PCS [46], PCNSC [56], NOP-CET [25]). Among the four instruments targeting multiple professional groups, two explicitly include care assistants (PCCF [63], SCIDS [59]). The four instruments targeting mixed nursing staff with various qualification levels, three explicitly include care assistants or care aides (PCS [46], PCNSC [56], NOP-CET [25]). The NOP-CET is a notable exception, offering adapted versions for assistant nurses, registered nurses, and nurse specialists [25]. In contrast, the remaining four instruments target licensed nurses exclusively, reflecting a more specialized, role-specific approach.
- **Variation by Setting**
  For instruments with specific competency foci, variation was observed across both development and application contexts. No single competency focus could be unequivocally attributed to a specific care setting. Three instruments (person-centered care: ICS-Nurse [61]; care of older people: KOP-Q [49]; dementia care: DCCS [47]) were applied in acute inpatient settings, whereas five instruments were used in LTC (palliative care: PCCF [63], BPW [57], PCS [46]; gerontology: GNC Scale [58]; care of older people: NOP-CET [25]). Two instruments were implemented across both acute inpatient and LTC settings (dementia care: SCIDS [59]; palliative care: PCNSC [56]). The P-CAT was originally developed for LTC settings [62], but the Korean version (K-P-CAT) has also been applied in geriatric hospitals [64]. The PCC Scale was developed and used in hospitals [60]. The Finnish version (PCC-Fin) was evaluated and used in LTC units and home care [65].

### Mode of data collection

Most instruments rely on self-reported ratings, reflecting a widespread use of self-evaluation for competence development and continuing professional education (*n* = 18). External assessment was used in two studies through comparison with external evaluations. The NCS was used to compare self-rated competence with supervisor ratings, revealing significant differences in perceived competence levels, which highlights the potential for self-assessment to underestimate or overestimate actual performance. Nurses and managers both rated competence as good, but managers scored higher. Correlations were weak, suggesting limited agreement. Social desirability bias may have influenced responses, potentially inflating scores and reducing validity [22]. Similarly, HNCS (short version) was employed to compare self-assessments with those of peers and supervisors, aiming to investigate the factors influencing agreement or disagreement between self- and external evaluations. The findings of this study suggest that aligning self- and external assessments requires shared perceptions of competence. Informal peer review in daily practice can facilitate this by enabling nurses to observe colleagues, reflect on feedback, and refine their self-appraisal, supporting continuous professional development [66].

### Psychometric properties of the instruments

Most instruments underwent systematic psychometric evaluation, including internal consistency (*n* = 20), content validity (*n* = 10), and construct validity (*n* = 18).

The psychometric evaluation of the instruments was highly uneven, with reliability and validity assessed in different ways across studies. This section does not aim to provide an exhaustive account of all measurements undertaken for each instrument. As such, we prioritized assessing the most commonly reported properties based on the available evidence: Reliability (e.g., Cronbach’s α, test–retest reliability), Construct validity (e.g., exploratory and confirmatory factor analysis), Content validity (e.g., Content Validity Index, expert review) and Cross-cultural validity [67]. As such, the primary aim is to map the existing evidence and provide a comprehensive overview of the types of instruments, their psychometric properties, and their applicability across contexts – not to conduct a critical appraisal of study quality or risk of bias.

#### Reliability

All 20 instruments reported internal consistency, with Cronbach’s α being the most commonly used measure. However, Cronbach’s α was not reported consistently across studies. Some articles reported values only for the overall scale, while others reported reliability coefficients only for the subscales. In some studies, both overall and subscale values were given. In Table 3, the overall Cronbach’s α is reported for all instruments. When overall values were not available, the range of subscale α-values is shown to reflect variability in internal consistency across domains.

**Table 3:** Internal consistency and stability of competency assessment instruments: Cronbach’s α and test–retest reliability

Cronbach’s α (*n* = 20)	
BPW (α = 0.71–0.84) [57]	NOP-CT (α = 0.516–0.93) [25]
DCCS (α = 0.73) [68], (α = 0.85–0.94) [47]	NPC (α = 0.97, 88-item scale) [54], (α = 0.97, short version) [69]
ENA (α = 0.77–0.93) [48]	P-CAT (α = 0.84) [62], K-P-CAT (α = 0.78) [64]
Ethan Questionnaire Tool (UK α = 0.971, Belgium α = 0.975, Germany α = 0.959, Greece α = 0.946, Spain α = 0.961, All Countries α = 0.963) [52]	PCS (α = 0.75–0.81) [46]
GNC Scale (α = 0.991) [58]	PCCF (α = 0.881–0.934) [63], (α = 0.862–0.959, for physicians) [55]
HNCS (α = 0.967) [45], (α = 0.93–0.95) [70], HNCS (short version, α = 0.94) [66]	PCC Scale (α = 0.94) [60] (α = 0.92) [71], PCC-Finn (α = 0.93) [65]
ICS-Nurse (α = 0.88–0.90) [61]	PCNSC (Reliability reported as high, no value reported) [56]
KOP-Q (α = 0.94) [49]	ProffNurse SAS (α = 0.772–0.940) [3], ProffNurse SAS II (α = 0.68–0.92) [72]
LTCNC Scale (α = 0.95–0.98) [51]	SCIDS (α = 0.91) [59], SCIDS-C (α = 0.87) [73]
NCCHS (α = 0.77–0.92) [50]	
**Test–retest reliability**,** Intraclass Correlation Coefficient (ICC) (n = 4)**:	
DCCS (ICC = 0.818) [47, 68]	P-CAT (ICC= value not reported) [62]
GNC Scale (ICC = 0.932) [58]	SCIDS (ICC = 0.74) [59], SCIDS-C (ICC = 0.88) [73]

A Cronbach’s α greater than 0.7 is generally considered indicative of acceptable reliability [67, 74]. The majority of reported values met or exceeded this threshold, reflecting good internal consistency across most instruments.

Notable exceptions were observed. The NOP-CET demonstrated good internal consistency across all three subscales—knowledge, skills, and personal attributes—although 10 of the 28 factors did not meet the α > 0.7 threshold. This limitation was attributed to the small number of items per scale (ranging from 2 to 4), which is commonly associated with reduced internal consistency. The authors further argued that, for complex psychological constructs such as competence, lower reliability coefficients are expected due to the heterogeneity of underlying subcomponents [25]. Unclear remains the relationship between the stated limitations and the use of different versions for Assistant Nurses, Registered Nurses, and Nurse Specialist [25].

The ETHAN Questionnaire Tool underwent psychometric validation across five European countries—the United Kingdom, Germany, Spain, Greece, and Belgium. Of the 40 reliability coefficients reported across eight domains, all but two, both derived from the Spanish dataset, exceeded 0.7, indicating satisfactory internal consistency in the other participating countries and within most domains in Spain. Moreover, the overall Cronbach’s alpha for the Spanish version was 0.961. Cronbach’s alpha coefficients for the pooled dataset from all partner countries also remained consistently above 0.7, supporting the instrument’s satisfactory internal consistency [52].

The PCCF was originally developed for physicians but was later adapted for nurses. Cronbach’s α values were high in both groups: 0.881–0.934 for nurses and healthcare assistants [63] and 0.862–0.959 for physicians [55]. While these values indicate good internal consistency, they do not, by themselves, confirm successful cross-professional transfer. The instrument’s content validity, construct validity, and item relevance for nurses and healthcare assistants must also be evaluated. The study by White, Agbana et al. reported that the PCCF was understood and applicable in nursing contexts, but no detailed validation of the instrument’s structure or item relevance for nurses was provided [63]. Therefore, while the high reliability suggests potential usability, further validation is needed to confirm that the PCCF measures the same construct in nurses as it does in physicians.

Test–retest reliability was assessed in 4 instruments, with Intraclass Correlation Coefficients (ICC) ranging from 0.74 to 0.932, indicating low random error variability and good stability over time [67] (Table 3).

#### Construct validity

Construct validity refers to the extent to which an instrument measures the construct under study. Construct validity was assessed using exploratory factor analysis (EFA) or confirmatory factor analysis (CFA), with EFA used to identify the underlying structure of the constructs and evaluate item clustering, and CFA employed to test the fit of the hypothesized factor structure against the observed data [67].

The reporting of results varied significantly across studies. While all studies reported at least adequate construct validity, the level of detail in reporting fit indices (e.g., CFI, RMSEA, TLI) was inconsistent. As a result, no specific values are reported here, only the methodological approach is described.

The following approaches to construct validity were used: in 6 studies, EFA was employed exclusively, specifically for the ENA [48], Ethan Questionnaire Tool [70], GNC Scale [58], NPC Scale [54], PCC [60] and the ProfNurse SAS [3]. In 6 studies, CFA was used exclusively for the HNCS [45], NCS [53, 75], PCNSC [56], PCS [46] and the SCIDS-C [73]. One study combined EFA and CFA for the PCC-Fin [65]. In 5 studies, factor analysis was reported without specifying the method, specifically for the ICS-Nurse [61], NOP-CET [25], NCCHS [50], P-CAT [62] and the SCIDS [59].

#### Content validity

Content validity refers to the extent to which an instrument’s items adequately represent the full range of the construct being measured [66].

In this review, content validity was reported in 10 studies. The instruments that reported content validity indices demonstrated consistently high values: SCIDS-C (Item-level Content Validity Index, I-CVI = 0.83–1.0, Scale-level index, S-CVI = 0.99) [73], DCCS (first version: S-CVI = 0.97, I-CVI = 0.94–0.98) [47], and the final version in another study (Content Validity Index - CVI = 0.97) [68], GNC Scale (CVI = 93.3%) [58], KOP-Q (S-CVI = 0.91) [49]. These values indicate strong expert consensus on item relevance and comprehensiveness [67]. Other instruments used different methods: the BPW employed a think-aloud method with experts [57], while the ICS-Nurse [61], HNCS [45], and NCS [53] were validated through pilot testing. The ETHAN questionnaire Tool underwent expert review in all partner countries (UK, Germany, Spain, Greece, Belgium) [52], and both the HNCS [45], and NCS [16], were validated through expert review.

#### Cross-cultural validity

Although several studies have examined the cross-cultural validity of competence assessment instruments, the following examples are presented to illustrate key findings rather than provide an exhaustive overview. Cross-cultural validity refers to the extent to which an instrument performs consistently across different cultural or linguistic contexts. The KOP-Q demonstrated cross-cultural validity between the Netherlands and the USA, with comparable results across both samples. This supports its use in educational and quality improvement programs in both countries, and allows for meaningful comparisons when using latent variable models [76]. However, cross-cultural validation should be repeated before use in other countries with different languages or cultures.

Structural validity refers to the extent to which the instrument’s structure reflects the underlying theoretical model. The NCS was evaluated for structural validity in multiple studies. A German validation (G-NCS) found that the original seven-factor model did not fit the data well; instead, a revised six-factor, 54-item structure demonstrated better model fit and internal consistency [75, 77]. This illustrates how established instruments may require structural modification to ensure cultural validity and reduce item redundancy or bias when implemented in diverse national settings.

### Summary of findings

This scoping review identified 20 competency assessment tools developed and validated across various professional roles and settings. The first research question aimed to map existing self-assessment tools for nursing competence, including their psychometric properties and target populations.

Self-assessment was the predominant data-collection method (*n* = 18), and all instruments demonstrated high reliability. While many demonstrated acceptable validity, methodological approaches varied considerably, limiting comparability and generalizability. Instruments were applied across different qualification levels and professional roles, supporting their potential use in diverse workforce structures, particularly relevant in LTC. Competency domains were highly heterogeneous, encompassing clinical, interpersonal, leadership, and context-specific competencies (e.g., dementia care, palliative care), underscoring the need for tools tailored to specific training or practice contexts.

The scope was intentionally broadened beyond residential LTC to include acute inpatient, community-based, and mixed-care settings. This reflects the transferability of core competencies across care environments and highlights the relevance of standardized assessment tools in multi-setting healthcare systems. However, the evidence base does not yet allow for definitive recommendations regarding the most effective instruments or their cross-context applicability.

The second research question explored the availability of validated instruments, their peer- or external-evaluation status, and their complementarity with self-assessment. Only two instruments incorporated a mixed-method approach combining self- and external evaluation. These dual-perspective tools offer a more comprehensive and objective assessment of competence, particularly in clinical, interpersonal, and leadership domains, thereby addressing potential biases inherent in self-reporting. This integration supports a more balanced and robust evaluation of professional capabilities.

Despite the availability of numerous self-assessment tools, a critical gap remains: no validated instruments specifically designed for healthcare assistants or care aides were identified. This absence underscores a significant limitation in the current evidence base and highlights an urgent need to develop and validate contextually appropriate, multi-rater assessment tools that reflect the realities of diverse nursing workforces and care environments.

## Discussion

This scoping review identified 20 instruments for assessing nursing competence across various qualification levels, care settings, and professional contexts. While many demonstrate strong psychometric properties, the findings reveal considerable variation in how competence is conceptualized, structured, and designed, highlighting a persistent lack of standardization in how competence is defined, measured, and applied. Most instruments focus on self-assessment among registered nurses, with limited inclusion of other qualification levels. Notably, no validated tools were found specifically designed for healthcare assistants or care aides, despite their central role in LTC.

The instruments range from brief, domain-specific scales to comprehensive, multidimensional ones. While some tools are customized for specific fields, such as dementia, palliative care or gerontology, few are designed for cross-sectoral or cross-cultural use. This restriction limits their complicates benchmarking across different settings. Nevertheless, competence assessment is increasingly seen as a strategic tool for workforce development, education, and quality improvement. Research projects like Germany’s KomPers demonstrate how validated instruments can inform staffing, supervision, and organizational learning in LTC settings, linking individual reflection with systemic change. To succeed, future tools must combine strong psychometric properties with practical usability, be sensitive to context, and be inclusive of the entire care workforce.

### Heterogeneity of competence conceptualizations

A central finding of this review is the substantial conceptual diversity in how “competence” is defined and operationalized across instruments. While general competence tools, such as the NCS [16] and the NPC Scale [54], are grounded in established theoretical frameworks like Benner’s novice-to-expert model, many specialized instruments were developed empirically for specific clinical roles or organizational needs.

This conceptual fragmentation is particularly evident in the LTC setting. Although general instruments like the NCS are promoted as applicable across healthcare settings, including LTC, most validation and implementation studies have been conducted in acute or clinical settings. Despite the distinct care demands of older adults with multimorbidity, dementia, and palliative needs, The transferability of these tools to long-term care is rarely documented. In contrast, specialized instruments, such as those for dementia care, palliative care or geriatric nursing, were developed with greater contextual specificity and thus demonstrate greater potential for applicability in LTC settings. However, even these tools lack consistent psychometric validation in LTC, and their use is highly heterogeneous across settings, with no single instrument clearly tied to one specific care context. This highlights the importance of targeted psychometric testing in residential LTC to improve the validity and reliability of assessments used in these environments.

Furthermore, instruments currently fail to adequately address emerging competence domains such as digital literacy, ethical decision-making, leadership and culturally sensitive care, despite these areas becoming increasingly important in modern LTC [1, 78, 79]. The ETHAN Questionnaire Tool [52] represents a promising step toward a holistic, cross-European competence framework integrating knowledge, skills, values, and attitudes, aligning with the study’s own holistic conception of competence [26, 27]. Yet, such integrative models remain rare.

### Predominance of self-assessment approaches

Self-assessment remains the dominant method in nursing competence instruments, reflecting a strong emphasis on reflective practice and professional self-awareness. While valuable for fostering personal insight and learning, self-report measures are inherently subjective and prone to biases such as overestimation, social desirability, and “faking good” [15–24]. Empirical evidence consistently shows limited agreement between self-rated and supervisor-rated competence, with nurses often overestimating their abilities, particularly in complex or high-stakes tasks [22, 66]. This discrepancy raises concerns about the validity of self-assessment when used for personnel decisions, staffing, or quality assurance in LTC, where accurate competence evaluation is critical for patient safety and care quality.

Notably, most instruments identified in this review were developed for research rather than for routine use in LTC practice. Despite their psychometric robustness, many are too lengthy (50–80 items) and lack practical feedback mechanisms, limiting their feasibility for daily supervision, staff development, or competence-based deployment. Only two tools integrate external assessment methods, such as supervisor ratings, despite evidence that multimodal approaches significantly enhance validity and objectivity [22, 66].

This overreliance on self-report reflects a broader methodological gap: competence is often treated as an internal, self-perceived construct rather than an externally verified performance standard. In the LTC context, characterized by high care complexity, staff shortages, and evolving care needs, this gap undermines the potential of competence assessment to support individual development, equitable staffing, and quality improvement. Future instruments must therefore prioritize practical usability, integration into routine workflows, and mixed-method designs that combine self-assessment with observation, testing, or supervisory ratings. Only such approaches can ensure that competence measurement is not only scientifically sound but also actionable, equitable, and meaningful in real-world LTC settings.

### Limited differentiation by qualification level

Despite the diverse qualification levels within the LTC workforce, ranging from registered nurses to healthcare assistants and support staff, most competence instruments fail to account for these professional hierarchies. While tools like the NOP-CET offer adapted versions for different roles, including assistant nurses, registered nurses, and specialists [25], such differentiation remains rare. Although many instruments are designed for use by multiple professional groups or mixed nursing teams with varying qualifications, they often administer the same questionnaire to all participants, despite significant differences in educational background, role-specific responsibilities and professional demands. This one-size-fits-all approach risks undermining the validity and relevance of competence assessments by failing to account for the distinct competencies required at different qualification levels. This lack of stratification undermines the potential for targeted competence development, equitable workforce planning, and qualification-sensitive staffing, key priorities in LTC, where care is delivered by multidisciplinary teams under high complexity and 24-hour demands [10, 11]. Notably, no validated competence instruments were identified in this review that are specifically designed for healthcare assistants, a group that constitutes a substantial and indispensable part of the LTC workforce. This absence is particularly concerning given their central role in daily care delivery.

The overrepresentation of tools focused on registered nurses reflects a broader gap in research and practice: the competence development needs of health care assistants remain systematically under-researched and under-supported. Without context-specific, validated instruments, their contributions are difficult to assess, recognize, or develop, limiting opportunities for professional growth, job satisfaction, and retention. In a sector facing persistent staffing shortages and high turnover, this represents a missed opportunity to strengthen the workforce through targeted, evidence-based development.

Future instrument development must prioritize qualification-sensitive design, with dedicated tools for healthcare assistants and other non-registered care staff. Such instruments should reflect the practical, task-oriented nature of their work while aligning with national qualification frameworks. Only by recognizing and assessing the entire spectrum of LTC nursing staff can competence measurement become genuinely inclusive, fair, and effective in supporting safe, professional care.

### Cultural and contextual limitations

The development and validation of competence instruments are strongly influenced by specific cultural and healthcare systems, predominantly in Scandinavia [16, 50, 54], Japan [45, 66], and China [80]. While some tools, such as GNC [58] and the SCIDS [73], have been adapted across countries, many remain tied to their original contexts, reducing their usefulness in different settings. Most instruments also target a single care sector, whether acute, long-term, or community care, despite the increasing demand for integrated care across transitions.

This sectoral and cultural fragmentation undermines the potential for cross-setting benchmarking and workforce planning. Although global frameworks like the ETHAN Questionnaire Tool [52] offer a promising route toward cross-national comparability, they often lack the detailed granularity needed for daily practice. Conversely, strong localized tools may not be easily transferable.

Therefore, future development of competence instruments should aim for a balance: creating universal frameworks that outline core competencies while developing context-specific tools for sector- and role-specific assessments. In LTC, where care is continuous, complex, and increasingly integrated with community services, adopting this dual approach is vital to ensuring both scientific validity and practical application.

### Validation and psychometric evidence

Robust instruments must demonstrate validity and reliability to allow justified inferences about performance levels [29]. Yet, despite growing interest, only a limited number of instruments meet these psychometric standards across diverse settings [30, 31]. Our review confirms that while many instruments exhibit promising psychometric properties, significant variability in methodological rigor persists. Some tools report only partial validation, and cross-cultural or cross-national validation remains scarce, raising concerns about their generalizability and interpretability—especially when used for high-stakes decisions such as personnel evaluation.

Notably, cross-national adaptations highlight both the potential and the challenges of international instrument transfer. For instance, the German validation of the Nurse Competence Scale (G-NCS) revealed that the original seven-factor structure did not replicate, necessitating a revised six-factor, 54-item model with improved fit and internal consistency [75, 77]. This underscores the critical importance of rigorous psychometric testing when adapting instruments across linguistic and healthcare systems, as cultural, organizational, and educational differences can introduce bias or reduce measurement accuracy. Instruments like the ETHAN Questionnaire Tool, which have demonstrated cross-national psychometric robustness, offer a promising model for international benchmarking and comparative research, addressing a key gap in current competence measurement [52].

However, the pursuit of international comparability should not overshadow the need for context-specific, practical tools. In LTC, where care needs are highly individualized and workflows are time-sensitive, overly abstract or lengthy instruments may be impractical. Concise, targeted tools that reflect real-world tasks and competencies are more likely to be adopted and effective for many applications, such as competence-based staffing, supervision, or continuous professional development. Future instrument development should therefore prioritize both psychometric rigor and contextual appropriateness, ensuring that assessments are not only scientifically sound but also meaningful and usable in daily LTC practice.

Moreover, the inclusion of secondary sources, such as adaptation studies and real-world implementation reports, enriched our understanding of how instruments are applied beyond their original contexts [58, 73, 81]. Yet, this also revealed a persistent gap: limited transparency in the original development, scaling, and full psychometric validation of many tools. However, in some cases, information regarding the original development or full psychometric validation of the instruments was limited, which may have affected the comparability of the findings. The lack of uniform reporting standards and limited guidance on score interpretation for competency assessments complicates benchmarking across studies and settings. These methodological inconsistencies underscore the need for greater transparency in the design, scaling, and reporting of competence-assessment instruments [67].

In conclusion, while many competence assessment tools demonstrate acceptable reliability and construct validity, the field remains fragmented by inconsistent validation practices and insufficient cross-cultural validation. To advance competence measurement in LTC, future research must focus on developing and validating concise, context-specific instruments with strong psychometric foundations, while simultaneously promoting international collaboration, standardized reporting, and systematic cross-national validation. Only through such efforts can we ensure that competence assessments are not only scientifically robust but also practically useful, equitable, and capable of informing high-quality, professional care in diverse LTC environments.

### Implications for practice, education, research, and policy

The absence of a universally valid competence instrument underscores the need for context-specific selection. In LTC, where care is complex, continuous, and delivered by a diverse workforce, tools must align with the target group, especially registered nurses, healthcare assistants, and care aides, and the intended purpose: individual development, staffing, or quality improvement.

For practice, competence assessment must shift from mere data collection to a strategic tool for leadership and workforce development. Nursing home managers play a pivotal role in embedding competence evaluation into routine supervision, staffing decisions, and professional development plans. By integrating validated instruments, such as those used in the KomPers project, into digital platforms or organizational workflows, managers can transform assessment results into actionable insights. This enables targeted training, equitable staffing, and transparent career pathways, ultimately strengthening team performance, staff retention, and care quality in LTC.

For education, instruments like the NPC or NCS can guide curriculum design and learning evaluation, provided that they are transparent and aligned with LTC-specific competencies. Specialized tools (e.g., DCCS, NCCHS) offer targeted support for dementia or palliative care training.

For research, future efforts must prioritize validating existing instruments in LTC settings, developing concise, modular tools for different qualification levels, especially for healthcare assistants, and integrating emerging competencies (e.g., digital literacy, ethical decision-making) into core frameworks.

For policymakers, these findings call for systemic investment in competence assessment as a foundation for nursing staff workforce sustainability. National and regional health authorities should support the development and validation of role-specific tools, integrate competence measurement into qualification frameworks, and promote cross-sectoral benchmarking. By embedding evidence-based assessment into policy, governments can ensure equitable staffing, improve care quality, and advance the professionalization of nursing in LTC.

Only by combining psychometric rigor with practical relevance can competence assessment become a meaningful driver of quality and professionalization in LTC.

### Limitations of the review

This scoping review does not claim to include every competence assessment tool developed globally. The search strategy targeted only peer-reviewed articles and pertinent grey literature in English and German. This ensured methodological consistency and reliable interpretation, using the research team’s language skills. However, limiting the search to these two languages reduces the generalizability of the results and might have excluded context-specific tools or studies published in other languages, especially from regions outside English- and German-speaking countries. Furthermore, focusing on assessment instruments rather than competence development, implementation, or contextual adaptation may have caused valuable research on how tools are used in practice, adapted across settings, or integrated into training or policy to be overlooked. Such studies may exist but were overlooked because they emphasize process, organizational change, or training rather than measurement tools.

Finally, much of the extracted information relied on the authors’ self-reported psychometric data, and not all validation results could be independently verified. Despite these limitations, this review provides a comprehensive synthesis of validated nursing competence assessment tools currently available in research and practice, offering a critical foundation for future instrument development and implementation in LTC.

## Conclusion

This scoping review addresses its two research questions directly. First, it identifies a variety of self-assessment instruments with sound psychometric properties that were primarily developed for registered nurses and validated in acute care settings. However, few tools differentiate by qualification level, and no validated instruments were found for healthcare assistants, revealing a critical gap in LTC workforce inclusivity. Second, there is a dearth of validated peer or external evaluation instruments, highlighting a strong reliance on self-perception and limited integration of objective assessment.

These findings highlight a persistent gap between research and practice in LTC. To bridge it, future instrument development must prioritize modular, theory-informed tools that are psychometrically robust, adaptable across roles and care sectors, and practical for daily use. Only by adopting context-sensitive and inclusive approaches can competence assessment effectively promote sustainability, quality, and professionalism in LTC.

## Supplementary Information

Below is the link to the electronic supplementary material.

Supplementary Material 1

Supplementary Material 2

## Data Availability

All data are available from the corresponding author upon reasonable request.

## Footnotes

1 Further information about the project is available at https://tcall.info/projekte/kompers/.

## Abbreviations

BPW: Bonner Palliative Knowledge Test
CINAHL: Cumulative Index to Nursing and Allied Health Literature
DCCS: Dementia care competence scale
ENA: Educational Needs Assessment
ETHAN: European Healthcare Training and Accreditation Network questionnaire
GNC scale: Gerontological nursing competencies scale
G-NCS: German Nurse Competence Scale
HNCS: Holistic nursing competence scale
ICN: International Council of Nurses
ICS-A-Nurse: Individualized Care Scale-Nurse
JBI: Joanna Briggs Institute
KOP-Q: Knowledge-about-Older-Patients-Quiz
LTC: Long-Term Care
LTCNC scale: The Long-Term Care nursing competences scale
NCCHS: Nurse Competence in Care Home Scale
NCS: Nurse Competence Scale
NOP-CET: Nursing Older People Competence Evaluation Tool
NPC: Nurse Professional Competence Scale
P-CAT: Person-Centered Care Assessment Tool
PCCF questionnaire: Palliative Care Competence Framework Questionnaire
PCC: Patient-centred Care Competency Scale
PCC-Fin: Finnish Patient-centred Care Competency Scale
PCNSC: Palliative Care Nursing Self-Competence Scale
PCS: Palliative Care Survey
PRISMA-ScR: Preferred Reporting Items for Systematic Reviews and Meta-Analyses Extension for Scoping Reviews
ProffNurse SAS II: Professional Nurse Self- Assessment Scale of clinical core competencies II
SCIDS: Sense of Competence in Dementia Care Staff

## References

[1] Koff SZ. Nursing in the European Union: Anatomy of a profession. Anderson: Routledge; 2017.

[2] Steven A, Tella S, Turunen H, Flores Vizcaya-Moreno M, Pérez-Cañaveras RM, Porras J, et al. Shared learning from national to international contexts: a research and innovation collaboration to enhance education for patient safety. J Res Nurs. 2019;24:149–64. 10.1177/1744987118824628. 34394520 10.1177/1744987118824628 PMC7932281

[3] Finnbakk E, Wangensteen S, Skovdahl K, Fagerström L. The Professional Nurse Self-Assessment Scale: Psychometric testing in Norwegian long term and home care contexts. BMC Nurs. 2015;14:59. 10.1186/s12912-015-0109-3. 26578847 10.1186/s12912-015-0109-3 PMC4647290

[4] Mei XX, Wang HY, Wu XN, Wu JY, Lu YZ, Ye ZJ. Self-Efficacy and Professional Identity Among Freshmen Nursing Students: A Latent Profile and Moderated Mediation Analysis. Front Psychol. 2022;13:779986. 10.3389/fpsyg.2022.779986. 35310284 10.3389/fpsyg.2022.779986 PMC8927723

[5] Eng C-J, Pai H-C. Determinants of nursing competence of nursing students in Taiwan: the role of self-reflection and insight. Nurse Educ Today. 2015;35:450–5. 10.1016/j.nedt.2014.11.021. 25534773 10.1016/j.nedt.2014.11.021

[6] Aiken LH, Clarke SP, Cheung RB, Sloane DM, Silber JH. Educational levels of hospital nurses and surgical patient mortality. JAMA. 2003;290:1617–23. 10.1001/jama.290.12.1617. 14506121 10.1001/jama.290.12.1617 PMC3077115

[7] Levett-Jones TL. Continuing education for nurses: a necessity or a nicety? J Contin Educ Nurs. 2005;36:229–33. 10.3928/0022-0124-20050901-10. 16218012 10.3928/0022-0124-20050901-10

[8] Hariyati RTS, Safril S. The relationship between nurses’ job satisfaction and continuing professional development. Enfermería Clínica. 2018;28:144–8. 10.1016/S1130-8621(18)30055-X.28073633

[9] Carlisle C, Luker KA, Davies C, Stilwell J, Wilson R. Skills competency in nurse education: nurse managers’ perceptions of diploma level preparation. J Adv Nurs. 1999;29:1256–64. 10.1046/j.1365-2648.1999.01011.x. 10320511 10.1046/j.1365-2648.1999.01011.x

[10] Aleo G, Pagnucci N, Walsh N, Watson R, Lang D, Kearns T, et al. The effectiveness of continuing professional development for the residential long-term care workforce: A systematic review. Nurse Educ Today. 2024;137:106161. 10.1016/j.nedt.2024.106161. 38493589 10.1016/j.nedt.2024.106161

[11] Specchia ML, Cozzolino MR, Carini E, Di Pilla A, Galletti C, Ricciardi W, Damiani G. Leadership Styles and Nurses’ Job Satisfaction. Results of a Systematic Review. Int J Environ Res Public Health. 2021. 10.3390/ijerph18041552. 33562016 10.3390/ijerph18041552 PMC7915070

[12] Faithfull S, Samuel C, Lemanska A, Warnock C, Greenfield D. Self-reported competence in long term care provision for adult cancer survivors: A cross sectional survey of nursing and allied health care professionals. Int J Nurs Stud. 2016;53:85–94. 10.1016/j.ijnurstu.2015.09.001. 26412775 10.1016/j.ijnurstu.2015.09.001

[13] Glerean N, Talman K, Glerean E, Hupli M, Haavisto E. Development and psychometric testing of the perception of nursing profession instrument. J Adv Nurs. 2023;79:4074–87. 10.1111/jan.15726. 37249182 10.1111/jan.15726

[14] Žiaková K, Kalánková D, Tomagová M. Assessing nurse professionalism: a literature review of instruments and their measurement properties. Cent Eur J Nurs Midw. 2022;13:611–23. 10.15452/cejnm.2021.12.0020.

[15] Minarik PA, Issue. Competence assessment and competency assurance of healthcare professionals. Clin Nurse Spec. 2005;19:180–3. 10.1097/00002800-200507000-00006. 16027544 10.1097/00002800-200507000-00006

[16] Meretoja R, Isoaho H, Leino-Kilpi H. Nurse competence scale: development and psychometric testing. J Adv Nurs. 2004;47:124–33. 10.1111/j.1365-2648.2004.03071.x. 15196186 10.1111/j.1365-2648.2004.03071.x

[17] Wangensteen S, Finnbakk E, Adolfsson A, Kristjansdottir G, Roodbol P, Ward H, Fagerström L. Postgraduate nurses’ self-assessment of clinical competence and need for further training. A European cross-sectional survey. Nurse Educ Today. 2018;62:101–6. 10.1016/j.nedt.2017.12.020. 29306748 10.1016/j.nedt.2017.12.020

[18] Baxter P, Norman G. Self-assessment or self deception? A lack of association between nursing students’ self-assessment and performance. J Adv Nurs. 2011;67:2406–13. 10.1111/j.1365-2648.2011.05658.x. 21517941 10.1111/j.1365-2648.2011.05658.x

[19] Freund PA, Kasten N. How smart do you think you are? A meta-analysis on the validity of self-estimates of cognitive ability. Psychol Bull. 2012;138:296–321. 10.1037/a0026556. 22181852 10.1037/a0026556

[20] Zell E, Krizan Z. Do People Have Insight Into Their Abilities? A Metasynthesis. Perspect Psychol Sci. 2014;9:111–25. 10.1177/1745691613518075. 26173249 10.1177/1745691613518075

[21] Bahreini M, Moattari M, Ahmadi F, Kaveh MH, Hayatdavoudy P, Mirzaei M. Comparison of head nurses and practicing nurses in nurse competence assessment. Iran J Nurs Midwifery Res. 2011;16:227–34. 22224112 PMC3249804

[22] Numminen O, Leino-Kilpi H, Isoaho H, Meretoja R. Congruence between nurse managers’ and nurses’ competence assessments: a correlation study. J Nurs Educ Pract. 2014. 10.5430/jnep.v5n1p142.

[23] John OP, Benet-Martínez V. Measurement: reliability, construct validation, and scale construction. In: Handbook of research methods in social and personality psychology. New York, NY, US: Cambridge University Press; 2000. pp. 339–69.

[24] Colthart I, Bagnall G, Evans A, Allbutt H, Haig A, Illing J, McKinstry B. The effectiveness of self-assessment on the identification of learner needs, learner activity, and impact on clinical practice: BEME Guide 10. Med Teach. 2008;30:124–45. 10.1080/01421590701881699. 18464136 10.1080/01421590701881699

[25] Bing-Jonsson PC, Hofoss D, Kirkevold M, Bjørk IT, Foss C. Nursing older people-competence evaluation tool: development and psychometric evaluation. J Nurs Meas. 2015;23:127–53. 10.1891/1061-3749.23.1.127. 25985500 10.1891/1061-3749.23.1.127

[26] Klieme E, Hartig J. Möglichkeiten und Voraussetzungen technologiebasierter Kompetenzdiagnostik. Eine Expertise im Auftrag des Bundesministeriums für Bildung und Forschung: Eine Expertise im Auftrag des BMBF. 2007. https://www.fachportal-paedagogik.de/literatur/vollanzeige.html?FId=3063878. Accessed 19 Apr 2026.

[27] Euler D. Kompetenzorientierung in der beruflichen Bildung. In: Arnold R, Lipsmeier A, Rohs M, editors. Handbuch Berufsbildung. 3rd ed. Wiesbaden: Springer VS; 2020. pp. 205–17. 10.1007/978-3-658-19312-6_18.

[28] Cowan DT, Norman I, Coopamah VP. Competence in nursing practice: a controversial concept–a focused review of literature. Nurse Educ Today. 2005;25:355–62. 10.1016/j.nedt.2005.03.002. 15904996 10.1016/j.nedt.2005.03.002

[29] Fitzpatrick R, Davey C, Buxton MJ, Jones DR. Evaluating patient-based outcome measures for use in clinical trials. Health Technol Assess. 1998;2:i–iv. 9812244

[30] Girot EA. Assessment of competence in clinical practice–a review of the literature. Nurse Educ Today. 1993;13:83–90. 10.1016/0260-6917(93)90023-u. 8502210 10.1016/0260-6917(93)90023-u

[31] Meretoja R, Leino-Kilpi H. Instruments for evaluating nurse competence. J Nurs Adm. 2001;31:346–52. 10.1097/00005110-200107000-00005. 11519263 10.1097/00005110-200107000-00005

[32] Spasova S, Baeten R, Coster S, Ghailani D, Peña-Casas R, Vanhercke B. Challenges in long-term care in Europe: a study of national policies 2018. Brussels: European Commission; 2018.

[33] von Elm E, Schreiber G, Haupt CC. Methodische Anleitung für Scoping Reviews (JBI-Methodologie). [Not Available]. Z Evid Fortbild Qual Gesundhwes. 2019;143:1–7. 10.1016/j.zefq.2019.05.004. 31296451 10.1016/j.zefq.2019.05.004

[34] Peters MDJ, Godfrey C, McInerney P, Munn Z, Tricco AC, Khalil H. Scoping reviews. In: Aromataris E, Lockwood C, Porritt K, Pilla B, Jordan Z, editors. JBI Manual for Evidence Synthesis. JBI; 2024. 10.46658/JBIMES-24-09.

[35] Munn Z, Peters MDJ, Stern C, Tufanaru C, McArthur A, Aromataris E. Systematic review or scoping review? Guidance for authors when choosing between a systematic or scoping review approach. BMC Med Res Methodol. 2018;18:143. 10.1186/s12874-018-0611-x. 30453902 10.1186/s12874-018-0611-x PMC6245623

[36] Tricco AC, Lillie E, Zarin W, O’Brien KK, Colquhoun H, Levac D, et al. PRISMA Extension for Scoping Reviews (PRISMA-ScR): Checklist and Explanation. Ann Intern Med. 2018;169:467–73. 10.7326/M18-0850. 30178033 10.7326/M18-0850

[37] Hirt J, Nordhausen T. Rechercheprotokoll für eine systematische Literaturrecherche. 2022. https://refhunter.org/research_support/rechercheprotokoll/. Accessed 5 Nov 2024.

[38] Arksey H, O’Malley L. Scoping studies: towards a methodological framework. Int J Soc Res Methodol. 2005;8:19–32. 10.1080/1364557032000119616.

[39] Peters MDJ, Marnie C, Tricco AC, Pollock D, Munn Z, Alexander L, et al. Updated methodological guidance for the conduct of scoping reviews. JBI Evid Implement. 2021;19:3–10. 10.1097/XEB.0000000000000277. 33570328 10.1097/XEB.0000000000000277

[40] Ouzzani M, Hammady H, Fedorowicz Z, Elmagarmid A. Rayyan-a web and mobile app for systematic reviews. Syst Rev. 2016;5:210. 10.1186/s13643-016-0384-4. 27919275 10.1186/s13643-016-0384-4 PMC5139140

[41] Fleiss JL. Measuring nominal scale, agreement among many raters. Psychol Bull. 1971;76:378–82.

[42] Landis JR, Koch GG. The Measurement of Observer Agreement for Categorical Data. Biometrics. 1977;33:159. 10.2307/2529310. 843571

[43] Mayring P. Qualitative Inhaltsanalyse Grundlagen und Techniken Philipp Mayring. Weinheim: Julius Beltz GmbH & Co. KG; 2022.

[44] Page MJ, McKenzie JE, Bossuyt PM, Boutron I, Hoffmann TC, Mulrow CD, et al. The PRISMA 2020 statement: an updated guideline for reporting systematic reviews. BMJ. 2021;372:n71. 10.1136/bmj.n71. 33782057 10.1136/bmj.n71 PMC8005924

[45] Takase M, Teraoka S. Development of the Holistic Nursing Competence Scale. Nurs Health Sci. 2011;13:396–403. 10.1111/j.1442-2018.2011.00631.x. 21883769 10.1111/j.1442-2018.2011.00631.x

[46] Thompson S, Bott M, Boyle D, Gajewski B, Tilden VP. A measure of palliative care in nursing homes. J Pain Symptom Manage. 2011;41:57–67. 10.1016/j.jpainsymman.2010.03.016. 20797836 10.1016/j.jpainsymman.2010.03.016 PMC3027846

[47] Yang Y-Y, Yang Y-P, Hsiao C-H, Kuo H-Y, Wang J-J. Development and psychometric testing of a dementia care competence scale for nurses working in acute care setting. Scand J Caring Sci. 2021;35:1179–86. 10.1111/scs.12936. 33368467 10.1111/scs.12936

[48] Kim M, Dyck MJ, Funk A. Initial Evidence for the Reliability and Validity of the Educational Needs Assessment Questionnaire. J Nurs Meas. 2016;24:442–53. 10.1891/1061-3749.24.3.442. 28714449 10.1891/1061-3749.24.3.442

[49] Dikken J, Hoogerduijn JG, Kruitwagen C, Schuurmans MJ. Content Validity and Psychometric Characteristics of the Knowledge about Older Patients Quiz for Nurses Using Item Response Theory. J Am Geriatr Soc. 2016;64:2378–83. 10.1111/jgs.14476. 27627575 10.1111/jgs.14476

[50] Kiljunen O, Partanen P, Välimäki T, Kankkunen P. Older people nursing in care homes: An examination of nursing professionals’ self-assessed competence and its predictors. Int J Older People Nurs. 2019;14:e12225. 10.1111/opn.12225. 30729686 10.1111/opn.12225

[51] Hsieh P-L, Chen C-M. Long term care nursing competence and related factors among Taiwanese nurses: A national survey for those who completed the LTC training course. Geriatr Nurs. 2017;38:192–8. 10.1016/j.gerinurse.2016.10.010. 27866668 10.1016/j.gerinurse.2016.10.010

[52] Cowan DT, Wilson-Barnett DJ, Norman IJ, Murrells T. Measuring nursing competence: development of a self-assessment tool for general nurses across Europe. Int J Nurs Stud. 2008;45:902–13. 10.1016/j.ijnurstu.2007.03.004. 17451716 10.1016/j.ijnurstu.2007.03.004

[53] Flinkman M, Leino-Kilpi H, Numminen O, Jeon Y, Kuokkanen L, Meretoja R. Nurse Competence Scale: a systematic and psychometric review. J Adv Nurs. 2017;73:1035–50. 10.1111/jan.13183. 27731918 10.1111/jan.13183

[54] Nilsson J, Johansson E, Egmar A-C, Florin J, Leksell J, Lepp M, et al. Development and validation of a new tool measuring nurses self-reported professional competence–the nurse professional competence (NPC) Scale. Nurse Educ Today. 2014;34:574–80. 10.1016/j.nedt.2013.07.016. 23938092 10.1016/j.nedt.2013.07.016

[55] Connolly M, McLean S, Guerin S, Walsh G, Barrett A, Ryan K. Development and Initial Psychometric Properties of a Questionnaire to Assess Competence in Palliative Care: Palliative Care Competence Framework Questionnaire. Am J Hosp Palliat Care. 2018;35:1304–8. 10.1177/1049909118772565. 29730936 10.1177/1049909118772565

[56] Sawatzky R, Della Roberts, Russell L, Bitschy A, Ho S, Desbiens J-F, et al. Self-Perceived Competence of Nurses and Care Aides Providing a Palliative Approach in Home, Hospital, and Residential Care Settings: A Cross-Sectional Survey. Can J Nurs Res. 2021;53:64–77. 10.1177/0844562119881043. 31645110 10.1177/0844562119881043

[57] Pfister D, Müller M, Müller S, Kern M, Rolke R, Radbruch L. Validierung des Bonner Palliativwissenstests (BPW). [Validation of the Bonn test for knowledge in palliative care (BPW)]. Schmerz. 2011;25:643–53. 10.1007/s00482-011-1111-7. 22120918 10.1007/s00482-011-1111-7

[58] Ho M-H, Lee JJ, Joo JY, Bail K, Liu MF, Traynor V. Measuring gerontological nursing competencies among aged care nurses: Cultural adaptation and psychometric validation. Int J Older People Nurs. 2023;18:e12551. 10.1111/opn.12551. 37209303 10.1111/opn.12551

[59] Schepers AK, Orrell M, Shanahan N, Spector A. Sense of competence in dementia care staff (SCIDS) scale: development, reliability, and validity. Int Psychogeriatr. 2012;24:1153–62. 10.1017/S104161021100247X. 22340666 10.1017/S104161021100247X

[60] Hwang J-I. Development and testing of a patient-centred care competency scale for hospital nurses. Int J Nurs Pract. 2015;21:43–51. 10.1111/ijn.12220. 24219042 10.1111/ijn.12220

[61] Suhonen R, Gustafsson M-L, Katajisto J, Välimäki M, Leino-Kilpi H. Individualized care scale - nurse version: a Finnish validation study. J Eval Clin Pract. 2010;16:145–54. 10.1111/j.1365-2753.2009.01168.x. 20074300 10.1111/j.1365-2753.2009.01168.x

[62] Edvardsson D, Fetherstonhaugh D, Nay R, Gibson S. Development and initial testing of the Person-centered Care Assessment Tool (P-CAT). Int Psychogeriatr. 2010;22:101–8. 10.1017/S1041610209990688. 19631005 10.1017/S1041610209990688

[63] White L, Agbana S, Connolly M, Larkin P, Guerin S. Palliative care competencies and education needs of nurses and healthcare assistants involved in the provision of supportive palliative care. Int J Palliat Nurs. 2021;27:195–204. 10.12968/ijpn.2021.27.4.195. 34169741 10.12968/ijpn.2021.27.4.195

[64] Huh A, Shin JH. Person-Centered Care Practice, Patient Safety Competence, and Patient Safety Nursing Activities of Nurses Working in Geriatric Hospitals. Int J Environ Res Public Health. 2021. 10.3390/ijerph18105169. 34068125 10.3390/ijerph18105169 PMC8152766

[65] Suhonen R, Lahtinen K, Stolt M, Pasanen M, Lemetti T. Validation of the Patient-Centred Care Competency Scale Instrument for Finnish Nurses. J Pers Med. 2021. 10.3390/jpm11060583. 34205569 10.3390/jpm11060583 PMC8235000

[66] Takase M, Yamamoto M, Sato Y. The factors related to self-other agreement/disagreement in nursing competence assessment: Comparative and correlational study. Int J Nurs Stud. 2018;80:147–54. 10.1016/j.ijnurstu.2018.01.011. 29426015 10.1016/j.ijnurstu.2018.01.011

[67] DeVon HA, Block ME, Moyle-Wright P, Ernst DM, Hayden SJ, Lazzara DJ, et al. A psychometric toolbox for testing validity and reliability. J Nurs Scholarsh. 2007;39:155–64. 10.1111/j.1547-5069.2007.00161.x. 17535316 10.1111/j.1547-5069.2007.00161.x

[68] Yang Y-Y, Hsiao C-H, Chang Y-J, Ma S-C, Wang J-J. Exploring dementia care competence of nurses working in acute care settings. J Clin Nurs. 2022;31:1972–82. 10.1111/jocn.15190. 31971304 10.1111/jocn.15190

[69] Halabi JO, Nilsson J, Lepp M. Professional Competence Among Registered Nurses Working in Hospitals in Saudi Arabia and Their Experiences of Quality of Nursing Care and Patient Safety. J Transcult Nurs. 2021;32:425–33. 10.1177/1043659621992845. 33576306 10.1177/1043659621992845

[70] Erdat Y, Kuruca-Ozdemir E, Kocoglu-Tanyer D, Duygulu S. The holistic nursing competence and transition shock of newly graduated nurses as the determinants of missed nursing care: The mediation analysis. J Clin Nurs. 2024;33:3576–85. 10.1111/jocn.17030. 38284458 10.1111/jocn.17030

[71] Pakkonen M, Stolt M, Edvardsson D, Pasanen M, Suhonen R. Person-centred care competence and person-centred care climate described by nurses in older people’s long-term care-A cross-sectional survey. Int J Older People Nurs. 2023;18:e12532. 10.1111/opn.12532. 36918384 10.1111/opn.12532

[72] Willman A, Bjuresäter K, Nilsson J. Newly graduated nurses’ clinical competencies and need for further training in acute care hospitals. J Clin Nurs. 2020;29:2209–20. 10.1111/jocn.15207. 32043711 10.1111/jocn.15207

[73] Zhao Y, Liu L, Ding Y, Shan Y, Chan HYL. Translation and validation of Chinese version of sense of competence in dementia care staff scale in healthcare providers: a cross-sectional study. BMC Nurs. 2022;21:35. 10.1186/s12912-022-00815-3. 35093043 10.1186/s12912-022-00815-3 PMC8801082

[74] Fayers PM, Machin D. Quality of life: Assessment, analysis and interpretation. Chichester, Weinheim: Wiley; 2000.

[75] Müller M. Nursing competence: psychometric evaluation using Rasch modelling. J Adv Nurs. 2013;69:1410–7. 10.1111/jan.12009. 22989303 10.1111/jan.12009

[76] Dikken J, Hoogerduijn JG, Klaassen S, Lagerwey MD, Shortridge-Baggett L, Schuurmans MJ. The Knowledge-about-Older-Patients - Quiz (KOP-Q) for nurses: Cross-cultural validation between the Netherlands and United States of America. Nurse Educ Today. 2017;55:26–30. 10.1016/j.nedt.2017.05.003. 28505522 10.1016/j.nedt.2017.05.003

[77] Girbig M, Bauer A. Kompetenzerfassung in der stationären Krankenpflege.: Übersetzung, Modizifierung und kulturelle Adaptation der Nurse Competence Scale (NCS). Pfle Wiss. 2011:655–63. 10.3936/1121.

[78] American Association of Colleges of Nursing. (2021). The essentials: Core competencies for professional nursing education. https://www.aacnnursing.org/Portals/42/AcademicNursing/pdf/Essentials-2021.pdf. Accessed 20 Apr 2026.

[79] Booth RG, Strudwick G, McBride S, O’Connor S, Solano López AL. How the nursing profession should adapt for a digital future. BMJ. 2021;n1190. 10.1136/bmj.n1190.

[80] Chen H, Pu L, Chen Q, Xu X, Bai C, Hu X. Instrument Development for Evaluation of Gerontological Nurse Specialists Core Competencies in China. Clin Nurse Spec. 2019;33:217–27. 10.1097/NUR.0000000000000469. 31404000 10.1097/NUR.0000000000000469

[81] Kada O, Janig H, Pinter G, Cernic K, Likar R. Palliativversorgung in Pflegeheimen: Ergebnisse einer Befragung zu Wissen und Selbstwirksamkeitserwartung von Pflegepersonal. [Palliative care in nursing homes: Results of a survey about knowledge and self-efficacy of nursing staff]. Schmerz. 2017;31:383–90. 10.1007/s00482-016-0184-8. 28078441 10.1007/s00482-016-0184-8

