# Supplementary material for: Nursing staff competence assessment instruments: a scoping review with implications for long-term care
Source: BMC Nurs. 2026 May 16;25:455. doi: 10.1186/s12912-026-04755-0 (PMC13185338; doi:10.1186/s12912-026-04755-0)
Supplement: Supplementary file 1 — Supplementary Material 1 [file 12912_2026_4755_MOESM1_ESM.docx]

Supplementary Table 1. Study designs and country of application for the 20 nursing competence assessment instruments included in this scoping review.

| **No** | **Name of Instrument** | **Included Articles (First Author/Versions with Context Information)** | **Study Design** | **Country** |
| --- | --- | --- | --- | --- |
| 1 | Nursing Older People Competence Evaluation Tool (NOP-CET) | Bing-Jonsson, Hofess et al. (2014) [1] | Development and Psychometric Evaluation | Norway |
| 2 | Educational Needs Assessment (ENA) | Kim, Dyck et al. (2016) [2] | Psychometric Evaluation, cross-sectional study | USA |
| 3 | Bonner Palliative Knowledge Test (BPW) | Pfister, Müller et al. (2011) [3] | Validation study | Germany |
|  |  | Kada, Janig et al. (2017) - palliative care competence, registered nurses [4] | cross-sectional study | Austria |
| 4 | Person- Centered Care Assessment Tool (P-CAT) | Edvardsson, Fetherstonhaugh et al. (2010) [5] | Development and initial testing | Australia |
|  |  | Huh, Shin (2021) - factors examined that influence patient safety in nursing activities (Used the Korean version K-P-CAT) [6] | descriptive survey design | Korea |
| 5 | Nurse Competence in Care Home Scale (NCCHS) | Kiljunen, Partanen et al. (2019) [7] | cross‐sectional survey | Finland |
| 6 | The Long-Term Care Nursing Competence Scale (LTCNC scale) | Hsieh, Chen (2017) [8] | cross-sectional study | Taiwan |
| 7 | Gerontological Nursing Competencies Scale (GNC) | Ho, Lee et al. - from a comprehensive gerontological nursing competency scale from Australia (2023) [9] | Translation and Validation | Taiwan |
| 8 | Palliative Care Survey (PCS) | Thompson, Bott et al. (2011) [10] | Psychometric Evaluation | USA |
|  |  | Smets, Pivodic et al. (2018) - cross-cultural adaptations [11] | cross-sectional study using a structured survey | Belgium, the Netherlands, England, Finland, Poland, Italy |
| 9 | Patient-centered Care Competency (PCC) Scale | Hwang (2015) [12] | cross-sectional survey design  Development and Psychometric Evaluation | South Korea |
|  |  | Hwang, Kim et al. (2019) investigate the degree of patient participation in patient safety activities in hospitals and its relationship to patient-centered care competence [13] | Cross-sectional study | South Korea |
|  |  | Lahtinen, Limetti et al. (2023) evaluate the perception of competence among registered nurses [14] | Descriptive correlative survey | Finland |
|  |  | Suhonen, Lahtinen et al. (2021) -Finnish version (PCC-Fin) [15] | Validation study | Finland |
|  |  | Pakkonen, Stolt et al. (2023) - analyse person-centred care competence and the person-centred care climate [16] | Descriptive cross-sectional survey design with cluster sampling | Finland |
| 10 | Individualized Care Scale-Nurse (ICS-Nurse) | Suhonen, Gustafsson et al. (2010) [17] | Methodological design | Finland |
|  |  | Lahtinen, Lemetti et al. (2023) - evaluate the perception of competence among registered nurses (used ICS-A-Nurse) [14] | Descriptive correlative survey | Finland |
| 11 | Knowledge-about-Older-Patients-Quiz (KOP-Q) | Dikken, Hoogerduijn et al. (2016) [18] | Cross-sectional design | The Netherlands |
|  |  | Dikken, Hoogerduijn et al. (2017b) - Crosscultural  validation [19] | Multicenter international cross-sectional design | USA, The Netherlands |
|  |  | Dikken, Hoogerduijn et al. (2017a) -Measurement of nurses’ attitudes and knowledge [20] | Multicenter cross sectional design | USA |
| 12 | Professional Nurse Self- Assessment Scale of Clinical Core Competencies (ProffNurse SAS) | Finnbakk, Wangensteen et al. (2015) [21] | Development and Psychometric Evaluation | Norway |
|  |  | Willman, Bjuresäter et al. (2020) - clinical competence and the need for further training (ProffNurse SAS II) [22] | Cross-sectional design | Sweden |
| 13 | Dementia Care Competence Scale (DCCS) | Yang, Yang et al. (2021) [23] | Instrument development design | Taiwan |
|  |  | Yang, Hsiao et al. (2022) [24] | One-sample descriptive-correlation design | Taiwan |
| 14 | Holistic Nursing Competence Scale (HNCS) | Takase, Teraoka (2011) [25] | Scale development | Japan |
|  |  | Takase, Yamamoto et al (2018) - short version HNCS [26] | Cross-sectional survey design (used HNCS short version) | Japan |
|  |  | Erdat, Kuruca-Ozdemir et al. (2024) - investigate the determinants of missed nursing care [27] | Descriptive and correlational design | Turkey |
| 15 | Nurse Professional Competence (NPC) Scale | Nilsson, Johansson et al. (2014) [28] | Methodological study Psychometric Evaluation | Sweden |
|  |  | Halabi, Nilsson et al. (2021) - questionnaire adopted for nurses in the Kingdom of Saudi Arabia (using short version of NPC) [29] | Cross-sectional design | Kingdom of Saudi Arabia |
| 16 | Nurse Competence Scale (NCS) | Meretoja, Isoaho et al. (2004) [30] | Development and psychometric evaluation | Finland |
|  |  | Girbig, Bauer (2011) -Translation into the German Version (G-NCS) [31] | Translation study | Germany |
|  |  | Müller (2012) -psychometric properties and evaluate the G-NCS [32] | Cross-sectional survey  psychometric evaluation | Switzerland |
|  |  | Numminen, Leino-Kilpi et al. (2014) - compared nurses’ and their managers’ competence assessments in Finland [33] | Cross-sectional, descriptive, comparative correlation design | Finland |
|  |  | Meretoja, Numminen et al. (2015) - compared nurse competence between three generational cohorts comprising the current nursing workforce. [34] | Cross-sectional study and descriptive, comparative design | Finland |
|  |  | Karlstedt, Wadensten et al. (2015) - training and self-assessed competence of Registered Nurses, geriatric care (used Swedish version of NCS) [35] | Cross-sectional, quantitative, descriptive, and explorative design. | Sweden |
|  |  | Lima, Newall et al. (2016) -Competence assessment, pediatric setting [36] | Longitudinal study | Australia |
|  |  | Flinkman, Leino-Kilpi (2017) - systematic and psychometric review of the NCS [37] | Systematic and psychometric review of the NCS | Multiple countries |
|  |  | Lahtinen, Lemetti et al. (2023) -competence-assessment, registered nurses [14] | Descriptive correlative survey | Finland |
| 17 | Palliative Care Nursing Self-Competence Scale (PCNSC) | Sawatzky, Roberts et al. (2021) [38] | Exploratory study design involved a correlational  analysis of cross-sectional survey data | Canada |
| 18 | Sense of Competence in Dementia Care Staff (SCIDS) | Schepers, Orrell, et al. (2012) [39] | Development and psychometric evaluation | United Kingdom |
|  |  | Zhao, Liu et al. (2022) - Translation and validation of Chinese version (SCIDS-C) [40] | Cross-sectional translation and validation study | China |
| 19 | Palliative Care Competence Framework Questionnaire (PCCF) | Connolly, McLean et al. (2018) [41] | Development and psychometric evaluation | Ireland |
|  |  | White, Agbana et al. (2021) - adopted for nurses and healthcare assistants [42] | Mixed-methods study using a sequential  exploratory design | Ireland |
| 20 | European Healthcare Training and Accreditation Network (ETHAN) Questionnaire Tool | Cowan et al. (2008) [43] | Development self-assessment tool | United Kingdom, Belgium, Greece, Germany, Spain |

References

1. Bing-Jonsson PC, Hofoss D, Kirkevold M, Bjørk IT, Foss C. Nursing older people-competence evaluation tool: development and psychometric evaluation. J Nurs Meas. 2015;23:127–53. doi:10.1891/1061-3749.23.1.127.

2. Kim M, Dyck MJ, Funk A. Initial Evidence for the Reliability and Validity of the Educational Needs Assessment Questionnaire. J Nurs Meas. 2016;24:442–53. doi:10.1891/1061-3749.24.3.442.

3. Pfister D, Müller M, Müller S, Kern M, Rolke R, Radbruch L. Validierung des Bonner Palliativwissenstests (BPW). [Validation of the Bonn test for knowledge in palliative care (BPW)]. Schmerz. 2011;25:643–53. doi:10.1007/s00482-011-1111-7.

4. Kada O, Janig H, Pinter G, Cernic K, Likar R. Palliativversorgung in Pflegeheimen : Ergebnisse einer Befragung zu Wissen und Selbstwirksamkeitserwartung von Pflegepersonal. [Palliative care in nursing homes : Results of a survey about knowledge and self-efficacy of nursing staff]. Schmerz. 2017;31:383–90. doi:10.1007/s00482-016-0184-8.

5. Edvardsson D, Fetherstonhaugh D, Nay R, Gibson S. Development and initial testing of the Person-centered Care Assessment Tool (P-CAT). Int Psychogeriatr. 2010;22:101–8. doi:10.1017/S1041610209990688.

6. Huh A, Shin JH. Person-Centered Care Practice, Patient Safety Competence, and Patient Safety Nursing Activities of Nurses Working in Geriatric Hospitals. Int J Environ Res Public Health 2021. doi:10.3390/ijerph18105169.

7. Kiljunen O, Partanen P, Välimäki T, Kankkunen P. Older people nursing in care homes: An examination of nursing professionals' self-assessed competence and its predictors. Int J Older People Nurs. 2019;14:e12225. doi:10.1111/opn.12225.

8. Hsieh P-L, Chen C-M. Long term care nursing competence and related factors among Taiwanese nurses: A national survey for those who completed the LTC training course. Geriatr Nurs. 2017;38:192–8. doi:10.1016/j.gerinurse.2016.10.010.

9. Ho M-H, Lee JJ, Joo JY, Bail K, Liu MF, Traynor V. Measuring gerontological nursing competencies among aged care nurses: Cultural adaptation and psychometric validation. Int J Older People Nurs. 2023;18:e12551. doi:10.1111/opn.12551.

10. Thompson S, Bott M, Boyle D, Gajewski B, Tilden VP. A measure of palliative care in nursing homes. J Pain Symptom Manage. 2011;41:57–67. doi:10.1016/j.jpainsymman.2010.03.016.

11. Smets T, Pivodic L, Piers R, Pasman HRW, Engels Y, Szczerbińska K, et al. The palliative care knowledge of nursing home staff: The EU FP7 PACE cross-sectional survey in 322 nursing homes in six European countries. Palliat Med. 2018;32:1487–97. doi:10.1177/0269216318785295.

12. Hwang J-I. Development and testing of a patient-centred care competency scale for hospital nurses. Int J Nurs Pract. 2015;21:43–51. doi:10.1111/ijn.12220.

13. Hwang J-I, Kim SW, Chin HJ. Patient Participation in Patient Safety and Its Relationships with Nurses' Patient-Centered Care Competency, Teamwork, and Safety Climate. Asian Nurs Res (Korean Soc Nurs Sci). 2019;13:130–6. doi:10.1016/j.anr.2019.03.001.

14. Lahtinen K, Lemetti T, Stolt M, Katajisto J, Suhonen R. Nurse competence provides more individuality in the care of older hospitalized people. Nurs Open. 2023;10:3191–200. doi:10.1002/nop2.1569.

15. Suhonen R, Lahtinen K, Stolt M, Pasanen M, Lemetti T. Validation of the Patient-Centred Care Competency Scale Instrument for Finnish Nurses. J Pers Med 2021. doi:10.3390/jpm11060583.

16. Pakkonen M, Stolt M, Edvardsson D, Pasanen M, Suhonen R. Person-centred care competence and person-centred care climate described by nurses in older people's long-term care-A cross-sectional survey. Int J Older People Nurs. 2023;18:e12532. doi:10.1111/opn.12532.

17. Suhonen R, Gustafsson M-L, Katajisto J, Välimäki M, Leino-Kilpi H. Individualized care scale - nurse version: a Finnish validation study. J Eval Clin Pract. 2010;16:145–54. doi:10.1111/j.1365-2753.2009.01168.x.

18. Dikken J, Hoogerduijn JG, Kruitwagen C, Schuurmans MJ. Content Validity and Psychometric Characteristics of the "Knowledge about Older Patients Quiz" for Nurses Using Item Response Theory. J Am Geriatr Soc. 2016;64:2378–83. doi:10.1111/jgs.14476.

19. Dikken J, Hoogerduijn JG, Klaassen S, Lagerwey MD, Shortridge-Baggett L, Schuurmans MJ. The Knowledge-about-Older-Patients - Quiz (KOP-Q) for nurses: Cross-cultural validation between the Netherlands and United States of America. Nurse Educ Today. 2017;55:26–30. doi:10.1016/j.nedt.2017.05.003.

20. Dikken J, Hoogerduijn JG, Lagerwey MD, Shortridge-Baggett L, Klaassen S, Schuurmans MJ. Measurement of nurses' attitudes and knowledge regarding acute care older patients: Psychometrics of the OPACS-US combined with the KOP-Q. Geriatr Nurs. 2017;38:393–7. doi:10.1016/j.gerinurse.2017.01.001.

21. Finnbakk E, Wangensteen S, Skovdahl K, Fagerström L. The Professional Nurse Self-Assessment Scale: Psychometric testing in Norwegian long term and home care contexts. BMC Nurs. 2015;14:59. doi:10.1186/s12912-015-0109-3.

22. Willman A, Bjuresäter K, Nilsson J. Newly graduated nurses' clinical competencies and need for further training in acute care hospitals. J Clin Nurs. 2020;29:2209–20. doi:10.1111/jocn.15207.

23. Yang Y-Y, Yang Y-P, Hsiao C-H, Kuo H-Y, Wang J-J. Development and psychometric testing of a dementia care competence scale for nurses working in acute care setting. Scand J Caring Sci. 2021;35:1179–86. doi:10.1111/scs.12936.

24. Yang Y-Y, Hsiao C-H, Chang Y-J, Ma S-C, Wang J-J. Exploring dementia care competence of nurses working in acute care settings. J Clin Nurs. 2022;31:1972–82. doi:10.1111/jocn.15190.

25. Takase M, Teraoka S. Development of the Holistic Nursing Competence Scale. Nurs Health Sci. 2011;13:396–403. doi:10.1111/j.1442-2018.2011.00631.x.

26. Takase M, Yamamoto M, Sato Y. The factors related to self-other agreement/disagreement in nursing competence assessment: Comparative and correlational study. Int J Nurs Stud. 2018;80:147–54. doi:10.1016/j.ijnurstu.2018.01.011.

27. Erdat Y, Kuruca-Ozdemir E, Kocoglu-Tanyer D, Duygulu S. The holistic nursing competence and transition shock of newly graduated nurses as the determinants of missed nursing care: The mediation analysis. J Clin Nurs. 2024;33:3576–85. doi:10.1111/jocn.17030.

28. Nilsson J, Johansson E, Egmar A-C, Florin J, Leksell J, Lepp M, et al. Development and validation of a new tool measuring nurses self-reported professional competence--the nurse professional competence (NPC) Scale. Nurse Educ Today. 2014;34:574–80. doi:10.1016/j.nedt.2013.07.016.

29. Halabi JO, Nilsson J, Lepp M. Professional Competence Among Registered Nurses Working in Hospitals in Saudi Arabia and Their Experiences of Quality of Nursing Care and Patient Safety. J Transcult Nurs. 2021;32:425–33. doi:10.1177/1043659621992845.

30. Meretoja R, Isoaho H, Leino-Kilpi H. Nurse competence scale: development and psychometric testing. J Adv Nurs. 2004;47:124–33. doi:10.1111/j.1365-2648.2004.03071.x.

31. Girbig M, Bauer A. Kompetenzerfassung in der stationären Krankenpflege.: Übersetzung, Modizifierung und kulturelle Adaptation der Nurse Competence Scale (NCS). Pfle.Wiss. 2011:655–63. doi:10.3936/1121.

32. Müller M. Nursing competence: psychometric evaluation using Rasch modelling. J Adv Nurs. 2013;69:1410–7. doi:10.1111/jan.12009.

33. Numminen O, Leino-Kilpi H, Isoaho H, Meretoja R. Congruence between nurse managers’ and nurses’ competence assessments: A correlation study. JNEP 2014. doi:10.5430/jnep.v5n1p142.

34. Meretoja R, Numminen O, Isoaho H, Leino-Kilpi H. Nurse competence between three generational nurse cohorts: A cross-sectional study. Int J Nurs Pract. 2015;21:350–8. doi:10.1111/ijn.12297.

35. Karlstedt M, Wadensten B, Fagerberg I, Pöder U. Is the competence of Swedish Registered Nurses working in municipal care of older people merely a question of age and postgraduate education? Scand J Caring Sci. 2015;29:307–16. doi:10.1111/scs.12164.

36. Lima S, Newall F, Jordan HL, Hamilton B, Kinney S. Development of competence in the first year of graduate nursing practice: a longitudinal study. J Adv Nurs. 2016;72:878–88. doi:10.1111/jan.12874.

37. Flinkman M, Leino-Kilpi H, Numminen O, Jeon Y, Kuokkanen L, Meretoja R. Nurse Competence Scale: a systematic and psychometric review. J Adv Nurs. 2017;73:1035–50. doi:10.1111/jan.13183.

38. Sawatzky R, Della Roberts, Russell L, Bitschy A, Ho S, Desbiens J-F, et al. Self-Perceived Competence of Nurses and Care Aides Providing a Palliative Approach in Home, Hospital, and Residential Care Settings: A Cross-Sectional Survey. Can J Nurs Res. 2021;53:64–77. doi:10.1177/0844562119881043.

39. Schepers AK, Orrell M, Shanahan N, Spector A. Sense of competence in dementia care staff (SCIDS) scale: development, reliability, and validity. Int Psychogeriatr. 2012;24:1153–62. doi:10.1017/S104161021100247X.

40. Zhao Y, Liu L, Ding Y, Shan Y, Chan HYL. Translation and validation of Chinese version of sense of competence in dementia care staff scale in healthcare providers: a cross-sectional study. BMC Nurs. 2022;21:35. doi:10.1186/s12912-022-00815-3.

41. Connolly M, McLean S, Guerin S, Walsh G, Barrett A, Ryan K. Development and Initial Psychometric Properties of a Questionnaire to Assess Competence in Palliative Care: Palliative Care Competence Framework Questionnaire. Am J Hosp Palliat Care. 2018;35:1304–8. doi:10.1177/1049909118772565.

42. White L, Agbana S, Connolly M, Larkin P, Guerin S. Palliative care competencies and education needs of nurses and healthcare assistants involved in the provision of supportive palliative care. Int J Palliat Nurs. 2021;27:195–204. doi:10.12968/ijpn.2021.27.4.195.

43. Cowan DT, Wilson-Barnett DJ, Norman IJ, Murrells T. Measuring nursing competence: development of a self-assessment tool for general nurses across Europe. Int J Nurs Stud. 2008;45:902–13. doi:10.1016/j.ijnurstu.2007.03.004.
