# Supplementary material for: Nursing staff competence assessment instruments: a scoping review with implications for long-term care
Source: BMC Nurs. 2026 May 16;25:455. doi: 10.1186/s12912-026-04755-0 (PMC13185338; doi:10.1186/s12912-026-04755-0)
Supplement: Supplementary file 2 — Supplementary Material 2 [file 12912_2026_4755_MOESM2_ESM.docx]

Supplementary Table 2. Characteristics of the included competence instruments (n=20).

| **No** | **Name of Instrument** | **Author (First published)** | **Versions** | **No. Items/Data Collection** | **Competency Foci** | **Instrument Structure and Categories** | **Response Formats** | **Psycho-**  **metrics** | | **Target groups and Professional Roles** | **Settings and Fields of Application** |
| --- | --- | --- | --- | --- | --- | --- | --- | --- | --- | --- | --- |
|  |  |  |  |  |  |  |  | **Reliability** | **Validity** |  |  |
| **General professional competence foci** | | | | | | | | | | | |
| 1 | Educational Needs Assessment (ENA) | Kim, Dyck et al. (2016) [1] | - | 32 / self-assessment | perceived learning requirements | Clinical Problems,  Professional Issues,  Managerial Skills,  Nursing Process,  Quality Improvements,  Clinical Interventions | Five-point Likert scale and open-ended questions | + | + | nursing-home nurses | Long-term care |
| 2 | European Healthcare Training and Accreditation Network (ETHAN) Questionnaire Tool | Cowan, Wilson-Barnett et al. (2008) [2] | - | 108 / Self-assessment | general professional nursing competence | Assessment  Care delivery  Communication  Health promotion and Illness prevention  Personal and professional development  Professional and ethical practice  Research and development  Teamworking | Four-point Likert scale | + | + | registered nurses and nursing students | Hospital |
| 3 | Nurse Competence Scale (NCS) | Meretoja, Isoaho et al. (2004) [3] | Girbig, Bauer (2011) [4]  Müller (2012) [5]  Numminen, Leino-Kilpi et al. (2014) [6]  Meretoja, Numminen et al. (2015) [7]  Karlstedt, Wadensten et al. (2015) [8]  Lima, Newall et al. (2016) [9]  Flinkman, Leino-Kilpi (2017) [10]  Lahtinen, Limetti et al. (2023) [11] | 73 or 54 / self-assessment and supervisor comparison | general professional competence | Helping role,  Teaching/coaching,  Diagnostic functions,  Situation management,  Therapeutic interventions,  Quality assurance,  Work role | visual analogy scale format and four-point Likert scale | + | + | Registered Nurses | general hospital,  community and specialized units,  all healthcare settings |
| 4 | Nurse Competence in Care Home Scale (NCCHS) | Kiljunen, Partanen et al. (2019) [12] | - | 84 / self-assessment | Competence in Care Home | Ethical practice,  Observation, communication, interaction,  Networks and cooperation,  Clinical care,  Safety, health, and holistic well-being,  Group leadership and activities | Four-point Likert scale | + | + | nurses | Long-term care and residential care home |
| 5 | Nurse Professional Competence (NPC) Scale | Nilsson, Johansson et al. (2014) [13] | Halabi, Nilsson et al. (2021) [14] | 88 or 35 / self-assessment | Professional Competence | Nursing care,  Value-based nursing care,  Medical/technical care,  Teaching/ learning and support,  Documentation and information technology,  Legislation in nursing and safety planning,  Leadership in and development of nursing care,  Education and supervision of staff/studen­ts | Four-point Likert scale | + | + | graduating nursing students and practicing nurses | general hospital,  community and specialized units |
| 6 | Holistic Nursing Competence Scale (HNCS) | Takase, Teraoka (2011) [15] | Takase, Yamamoto et al. (2018) [16]  Erdat, Kuruca-Ozdemir et al. (2024) [17] | 36 or 12/ self-assessment and external assessment | Nursing competence | Part A: Aptitude,  Part B: Staff Education,  Management,  Ethically Oriented Practice,  Nursing Care in Team and Professional Development. | Seven-point Likert scale | + | + | registered nurses | acute care settings |
| 7 | The Long-Term Care Nursing Competence Scale (LTCNC Scale) | Hsieh, Chen (2017) [18] | - | 67 / self-assessment | clinical competence | Professional, ethical, and legal practice,  Care provision management,  Professional development | Four-point Likert scale | + | n.a. | nurses | Long-term care and residential environments |
| 8 | Professional Nurse Self- Assessment Scale of Clinical Core Competencies (ProffNurse SAS) | Finnbakk, Wangensteen et al. (2015) [19] | Willman, Bjuresäter et al. (2020) [20] | 74 or 50 / self-assessment | clinical core competencies and need for further training | Direct clinical practice,  Professional development,  Ethical decision-making,  Clinical leadership,  Cooperation and consultation,  Critical thinking | Ten-point Likert scale | + | + | registered nurses | acute care settings |
| **Specialized competence foci** | | | | | | | | | | | |
| 9 | Person- Centered Care Assessment Tool (P-CAT) | Edvardsson, Fetherstonhaugh et al. (2010) [21] | Huh, Shin (2021) [22] | 13 / self-assessment | Person-Centered Care | Person-centredness,  Environmental support | Five-point Likert scale | + | + | aged care staff | Long-term aged-care settings, geriatric hospitals |
| 10 | Patient-centred Care Competency (PCC) Scale | Hwang (2015) [23] | Hwang, Kim et al. (2019) [24]  Suhonen, Lahtinen et al. (2021) [25]  Lahtinen, Limetti et al. (2023) [11]  Pakkonen, Stolt et al. (2023) [26] | 17 / self-assessment | patient-centred competence | Including Respecting patients’ perspectives,  Promoting patient involvement in care processes,  Providing for patient comfort,  Advocating for patients | Five-point Likert scale | + | + | nurses | Hospital  Long-term care units and home care (PCC-Fin |
| 11 | Individualized Care Scale-Nurse (ICS-Nurse) | Suhonen, Gustafsson et al. (2010) [27] | Lahtinen, Limetti et al. (2023) [11] | 34 / self-assessment | Individualized Care | ICS-A-Nurse: Patients’ clinical situation,  Personal life situation,  Decisional control  ICS-B-Nurse: Clinical situation ,personal life situation, decisional control over care | Five-point Likert scale | + | + | Nurses | Inpatient wards |
| 12 | Palliative Care Competence Framework Questionnaire (PCCF) | Connolly, McLean et al. (2018) [28] | White, Agbana et al. (2021) [29] | 27 /self-assessment | palliative care | Knowledge,  Attitudes,  Behaviour | Five-point Likert scale | + | +  (Only for physi­cians) | physicians in Ireland (Connolly, McLean et al. 2018)  nurses and  healthcare assistants (White, Agbana et al. 2021) | residential care |
| 13 | Bonner Palliative Knowledge Test (BPW) | Pfister, Müller et al. (2011) [30] | Kada, Janig et al. (2017) [31] | 30 / self-assessment | Palliative Care | Knowledge,  Self-efficacy | Four-point Likert scale | +. | + | nursing-home staff | Nursing homes |
| 14 | Palliative Care Survey (PCS) | Thompson, Bott et al. (2011) [32] | Smets, Pivodic et al. (2018) [33] | 51 / self-assessment | Palliative Care Practice and Palliative Care Knowledge | Palliative Care Practice: bereavement,  planning/intervention,  communication with family,  provider coordination  Palliative Care Knowledge: psychological,  physical,  end-of-life factors. | Dichotomous scale and five-point Likert scale. | + | + | Nurses and care assistants | long-term care facility for older people |
| 15 | Palliative Care Nursing Self-Competence Scale (PCNSC) | Sawatzky, Roberts et al. (2021) [34] | - | 50 / self-assessment | palliative care competence | Physical needs, pain,  Physical needs, other symptoms,  Psychological needs,  Social needs,  Spiritual needs,  Needs related to functional status,  Ethical and legal issues,  Interprofessional collaboration and communication,  personal and professional issues related to nursing care  Last hours of life. | Four-point Likert scale. | + | + | nurses and care  aides | hospital, home, and residential settings |
| 16 | Gerontological Nursing Competencies (GNC) Scale | Ho, Lee et al. (2023) [35] | - | 33 / self-assessment | gerontological nursing competencies | Core 1: Living well for older people across communities and groups,  Core 2: Maximising health outcomes,  Core 3: Communication effectively,  Core 4: Facilitating transitions in care,  Core 5: Facilitating choices within legal an ethical framework,  Core 6: Partnering with family & carers,  Core 7: Promoting mental health an psychological well-being,  Core 8: Providing evidence-based dementia-care,  Core 9: Promoting optimal pain management,  Core 10: Providing palliative care,  Core 11: Enabling access to technology | Five-point Likert scale | + | + | aged care Registered Nurse | Nursing homes, long-term  care facilities and aged care wards in Taiwan |
| 17 | Knowledge-about-Older-Patients-Quiz (KOP-Q) | Dikken, Hoogerduijn et al. (2016) [36] | Dikken, Hoogerduijn et al. (2017a) [37]  Dikken, Hoogerduijn et al. (2017b) [38] | 30 / self-assessment | Knowledge about Older Patients | Knowledge about Older Patients | True or false | + | + | nursing students, hospital nurses, and geriatric specialists | Hospital |
| 18 | Nursing Older People Competence Evaluation Tool (NOP-CET) | Bing-Jonsson, Hofess et al. (2014) [39] | - | 65 (346 subitems)/self-assessment | Care for older people | Knowledge,  Skills,  Personal attributes | Likert scale and “True or false” questions with case references, partly knowledge-based | + | + | Registered nurses  Assistant nurses  assistants | Home care and residential care for older people |
| 19 | Dementia Care Competence Scale (DCCS) | Yang, Yang et al. (2021) [40] | Yang, Hsiao et al. (2022) [41] | 31 / self-assessment | dementia care competence | Knowledge subscale,  Skills subscale,  Attitude subscale | Likert scale and “True or false” questions. | + | + | nurses | acute care settings |
| 20 | Sense of Competence in Dementia Care Staff (SCIDS) | Schepers, Orrell, et al. (2012) [42] | Zhao, Liu et al. (2022) [43] | 17 / self-assessment | dementia-care competence | Professionalism,  Building Relationships,  Care Challenges,  Sustaining Personhood | Four-point Likert scale | + | + | healthcare providers  (care assistants, nurses, doctors, physiotherapists, and social workers) | nursing homes and hospital |

References

1. Kim M, Dyck MJ, Funk A. Initial Evidence for the Reliability and Validity of the Educational Needs Assessment Questionnaire. J Nurs Meas. 2016;24:442–53. doi:10.1891/1061-3749.24.3.442.

2. Cowan DT, Jenifer Wilson-Barnett D, Norman IJ, Murrells T. Measuring nursing competence: development of a self-assessment tool for general nurses across Europe. Int J Nurs Stud. 2008;45:902–13. doi:10.1016/j.ijnurstu.2007.03.004.

3. Meretoja R, Isoaho H, Leino-Kilpi H. Nurse competence scale: development and psychometric testing. J Adv Nurs. 2004;47:124–33. doi:10.1111/j.1365-2648.2004.03071.x.

4. Girbig M, Bauer A. Kompetenzerfassung in der stationären Krankenpflege.: Übersetzung, Modizifierung und kulturelle Adaptation der Nurse Competence Scale (NCS). Pfle.Wiss. 2011:655–63. doi:10.3936/1121.

5. Müller M. Nursing competence: psychometric evaluation using Rasch modelling. J Adv Nurs. 2013;69:1410–7. doi:10.1111/jan.12009.

6. Numminen O, Leino-Kilpi H, Isoaho H, Meretoja R. Congruence between nurse managers’ and nurses’ competence assessments: A correlation study. JNEP 2014. doi:10.5430/jnep.v5n1p142.

7. Meretoja R, Numminen O, Isoaho H, Leino-Kilpi H. Nurse competence between three generational nurse cohorts: A cross-sectional study. Int J Nurs Pract. 2015;21:350–8. doi:10.1111/ijn.12297.

8. Karlstedt M, Wadensten B, Fagerberg I, Pöder U. Is the competence of Swedish Registered Nurses working in municipal care of older people merely a question of age and postgraduate education? Scand J Caring Sci. 2015;29:307–16. doi:10.1111/scs.12164.

9. Lima S, Newall F, Jordan HL, Hamilton B, Kinney S. Development of competence in the first year of graduate nursing practice: a longitudinal study. J Adv Nurs. 2016;72:878–88. doi:10.1111/jan.12874.

10. Flinkman M, Leino-Kilpi H, Numminen O, Jeon Y, Kuokkanen L, Meretoja R. Nurse Competence Scale: a systematic and psychometric review. J Adv Nurs. 2017;73:1035–50. doi:10.1111/jan.13183.

11. Lahtinen K, Lemetti T, Stolt M, Katajisto J, Suhonen R. Nurse competence provides more individuality in the care of older hospitalized people. Nurs Open. 2023;10:3191–200. doi:10.1002/nop2.1569.

12. Kiljunen O, Partanen P, Välimäki T, Kankkunen P. Older people nursing in care homes: An examination of nursing professionals' self-assessed competence and its predictors. Int J Older People Nurs. 2019;14:e12225. doi:10.1111/opn.12225.

13. Nilsson J, Johansson E, Egmar A-C, Florin J, Leksell J, Lepp M, et al. Development and validation of a new tool measuring nurses self-reported professional competence--the nurse professional competence (NPC) Scale. Nurse Educ Today. 2014;34:574–80. doi:10.1016/j.nedt.2013.07.016.

14. Halabi JO, Nilsson J, Lepp M. Professional Competence Among Registered Nurses Working in Hospitals in Saudi Arabia and Their Experiences of Quality of Nursing Care and Patient Safety. J Transcult Nurs. 2021;32:425–33. doi:10.1177/1043659621992845.

15. Takase M, Teraoka S. Development of the Holistic Nursing Competence Scale. Nurs Health Sci. 2011;13:396–403. doi:10.1111/j.1442-2018.2011.00631.x.

16. Takase M, Yamamoto M, Sato Y. The factors related to self-other agreement/disagreement in nursing competence assessment: Comparative and correlational study. Int J Nurs Stud. 2018;80:147–54. doi:10.1016/j.ijnurstu.2018.01.011.

17. Erdat Y, Kuruca-Ozdemir E, Kocoglu-Tanyer D, Duygulu S. The holistic nursing competence and transition shock of newly graduated nurses as the determinants of missed nursing care: The mediation analysis. J Clin Nurs. 2024;33:3576–85. doi:10.1111/jocn.17030.

18. Hsieh P-L, Chen C-M. Long term care nursing competence and related factors among Taiwanese nurses: A national survey for those who completed the LTC training course. Geriatr Nurs. 2017;38:192–8. doi:10.1016/j.gerinurse.2016.10.010.

19. Finnbakk E, Wangensteen S, Skovdahl K, Fagerström L. The Professional Nurse Self-Assessment Scale: Psychometric testing in Norwegian long term and home care contexts. BMC Nurs. 2015;14:59. doi:10.1186/s12912-015-0109-3.

20. Willman A, Bjuresäter K, Nilsson J. Newly graduated nurses' clinical competencies and need for further training in acute care hospitals. J Clin Nurs. 2020;29:2209–20. doi:10.1111/jocn.15207.

21. Edvardsson D, Fetherstonhaugh D, Nay R, Gibson S. Development and initial testing of the Person-centered Care Assessment Tool (P-CAT). Int Psychogeriatr. 2010;22:101–8. doi:10.1017/S1041610209990688.

22. Huh A, Shin JH. Person-Centered Care Practice, Patient Safety Competence, and Patient Safety Nursing Activities of Nurses Working in Geriatric Hospitals. Int J Environ Res Public Health 2021. doi:10.3390/ijerph18105169.

23. Hwang J-I. Development and testing of a patient-centred care competency scale for hospital nurses. Int J Nurs Pract. 2015;21:43–51. doi:10.1111/ijn.12220.

24. Hwang J-I, Kim SW, Chin HJ. Patient Participation in Patient Safety and Its Relationships with Nurses' Patient-Centered Care Competency, Teamwork, and Safety Climate. Asian Nurs Res (Korean Soc Nurs Sci). 2019;13:130–6. doi:10.1016/j.anr.2019.03.001.

25. Suhonen R, Lahtinen K, Stolt M, Pasanen M, Lemetti T. Validation of the Patient-Centred Care Competency Scale Instrument for Finnish Nurses. J Pers Med 2021. doi:10.3390/jpm11060583.

26. Pakkonen M, Stolt M, Edvardsson D, Pasanen M, Suhonen R. Person-centred care competence and person-centred care climate described by nurses in older people's long-term care-A cross-sectional survey. Int J Older People Nurs. 2023;18:e12532. doi:10.1111/opn.12532.

27. Suhonen R, Gustafsson M-L, Katajisto J, Välimäki M, Leino-Kilpi H. Individualized care scale - nurse version: a Finnish validation study. J Eval Clin Pract. 2010;16:145–54. doi:10.1111/j.1365-2753.2009.01168.x.

28. Connolly M, McLean S, Guerin S, Walsh G, Barrett A, Ryan K. Development and Initial Psychometric Properties of a Questionnaire to Assess Competence in Palliative Care: Palliative Care Competence Framework Questionnaire. Am J Hosp Palliat Care. 2018;35:1304–8. doi:10.1177/1049909118772565.

29. White L, Agbana S, Connolly M, Larkin P, Guerin S. Palliative care competencies and education needs of nurses and healthcare assistants involved in the provision of supportive palliative care. Int J Palliat Nurs. 2021;27:195–204. doi:10.12968/ijpn.2021.27.4.195.

30. Pfister D, Müller M, Müller S, Kern M, Rolke R, Radbruch L. Validierung des Bonner Palliativwissenstests (BPW). [Validation of the Bonn test for knowledge in palliative care (BPW)]. Schmerz. 2011;25:643–53. doi:10.1007/s00482-011-1111-7.

31. Kada O, Janig H, Pinter G, Cernic K, Likar R. Palliativversorgung in Pflegeheimen : Ergebnisse einer Befragung zu Wissen und Selbstwirksamkeitserwartung von Pflegepersonal. [Palliative care in nursing homes : Results of a survey about knowledge and self-efficacy of nursing staff]. Schmerz. 2017;31:383–90. doi:10.1007/s00482-016-0184-8.

32. Thompson S, Bott M, Boyle D, Gajewski B, Tilden VP. A measure of palliative care in nursing homes. J Pain Symptom Manage. 2011;41:57–67. doi:10.1016/j.jpainsymman.2010.03.016.

33. Smets T, Pivodic L, Piers R, Pasman HRW, Engels Y, Szczerbińska K, et al. The palliative care knowledge of nursing home staff: The EU FP7 PACE cross-sectional survey in 322 nursing homes in six European countries. Palliat Med. 2018;32:1487–97. doi:10.1177/0269216318785295.

34. Sawatzky R, Della Roberts, Russell L, Bitschy A, Ho S, Desbiens J-F, et al. Self-Perceived Competence of Nurses and Care Aides Providing a Palliative Approach in Home, Hospital, and Residential Care Settings: A Cross-Sectional Survey. Can J Nurs Res. 2021;53:64–77. doi:10.1177/0844562119881043.

35. Ho M-H, Lee JJ, Joo JY, Bail K, Liu MF, Traynor V. Measuring gerontological nursing competencies among aged care nurses: Cultural adaptation and psychometric validation. Int J Older People Nurs. 2023;18:e12551. doi:10.1111/opn.12551.

36. Dikken J, Hoogerduijn JG, Kruitwagen C, Schuurmans MJ. Content Validity and Psychometric Characteristics of the "Knowledge about Older Patients Quiz" for Nurses Using Item Response Theory. J Am Geriatr Soc. 2016;64:2378–83. doi:10.1111/jgs.14476.

37. Dikken J, Hoogerduijn JG, Lagerwey MD, Shortridge-Baggett L, Klaassen S, Schuurmans MJ. Measurement of nurses' attitudes and knowledge regarding acute care older patients: Psychometrics of the OPACS-US combined with the KOP-Q. Geriatr Nurs. 2017;38:393–7. doi:10.1016/j.gerinurse.2017.01.001.

38. Dikken J, Hoogerduijn JG, Klaassen S, Lagerwey MD, Shortridge-Baggett L, Schuurmans MJ. The Knowledge-about-Older-Patients - Quiz (KOP-Q) for nurses: Cross-cultural validation between the Netherlands and United States of America. Nurse Educ Today. 2017;55:26–30. doi:10.1016/j.nedt.2017.05.003.

39. Bing-Jonsson PC, Hofoss D, Kirkevold M, Bjørk IT, Foss C. Nursing older people-competence evaluation tool: development and psychometric evaluation. J Nurs Meas. 2015;23:127–53. doi:10.1891/1061-3749.23.1.127.

40. Yang Y-Y, Yang Y-P, Hsiao C-H, Kuo H-Y, Wang J-J. Development and psychometric testing of a dementia care competence scale for nurses working in acute care setting. Scand J Caring Sci. 2021;35:1179–86. doi:10.1111/scs.12936.

41. Yang Y-Y, Hsiao C-H, Chang Y-J, Ma S-C, Wang J-J. Exploring dementia care competence of nurses working in acute care settings. J Clin Nurs. 2022;31:1972–82. doi:10.1111/jocn.15190.

42. Schepers AK, Orrell M, Shanahan N, Spector A. Sense of competence in dementia care staff (SCIDS) scale: development, reliability, and validity. Int Psychogeriatr. 2012;24:1153–62. doi:10.1017/S104161021100247X.

43. Zhao Y, Liu L, Ding Y, Shan Y, Chan HYL. Translation and validation of Chinese version of sense of competence in dementia care staff scale in healthcare providers: a cross-sectional study. BMC Nurs. 2022;21:35. doi:10.1186/s12912-022-00815-3.
